# A Commensal Bacterium Promotes Virulence of an Opportunistic Pathogen via Cross-Respiration

**DOI:** 10.1128/mBio.00782-16

**Published:** 2016-06-28

**Authors:** Apollo Stacy, Derek Fleming, Richard J. Lamont, Kendra P. Rumbaugh, Marvin Whiteley

**Affiliations:** a Department of Molecular Biosciences, Institute of Cellular and Molecular Biology, Center for Infectious Disease, The University of Texas at Austin, Austin, Texas, USA; b Department of Surgery, Texas Tech University Health Sciences Center, Lubbock, Texas, USA; c Department of Oral Immunology and Infectious Diseases, School of Dentistry, University of Louisville, Louisville, Kentucky, USA

## Abstract

Bacteria rarely inhabit infection sites alone, instead residing in diverse, multispecies communities. Despite this fact, bacterial pathogenesis studies primarily focus on monoculture infections, overlooking how community interactions influence the course of disease. In this study, we used global mutant fitness profiling (transposon sequencing [Tn-seq]) to determine the genetic requirements for the pathogenic bacterium *Aggregatibacter actinomycetemcomitans* to cause disease when coinfecting with the commensal bacterium *Streptococcus gordonii*. Our results show that *S. gordonii* extensively alters *A. actinomycetemcomitans* requirements for virulence factors and biosynthetic pathways during infection. In addition, we discovered that the presence of *S. gordonii* enhances the bioavailability of oxygen during infection, allowing *A. actinomycetemcomitans* to shift from a primarily fermentative to a respiratory metabolism that enhances its growth yields and persistence. Mechanistically, respiratory metabolism enhances the fitness of *A. actinomycetemcomitans in vivo* by increasing ATP yields via central metabolism and creating a proton motive force. Our results reveal that, similar to cross-feeding, where one species provides another species with a nutrient, commensal bacteria can also provide electron acceptors that promote the respiratory growth and fitness of pathogens *in vivo*, an interaction that we term cross-respiration.

## IMPORTANCE

Commensal bacteria can enhance the virulence of pathogens in mixed-species infections. However, knowledge of the mechanisms underlying this clinically relevant phenomenon is lacking. To bridge this gap, we comprehensively determined the genes a pathogen needs to establish coinfection with a commensal. Our findings show that the metabolism of the pathogen is low-energy-yielding in monoinfection, but in coinfection, the commensal improves the fitness of the pathogen by increasing the bioavailability of oxygen, thereby shifting the pathogen toward a high-energy-yielding metabolism. Similar to cross-feeding, this interaction, which we term cross-respiration, illustrates that commensal bacteria can provide electron acceptors that enhance the virulence of pathogens during infection.

## INTRODUCTION

The history of microbiology is marked by discoveries of the causative agents of devastating human infections. In many cases, such as cholera (1) and anthrax (2), the causative agent is a single pathogen, which can be demonstrated using Koch’s postulates. While studies of pathogens are often focused on how they overcome host-associated stresses and the immune system, it is also clear that to establish an infection, pathogens must also compete with the host microbiome for space and nutrients. This is especially clear for infections of the large intestine (3, 4), as this organ is home to a stable microbiome (5). Many other human infections are also polymicrobial in nature, such as wounds (6), abscesses (7), and the lungs of cystic fibrosis patients (8). Interspecies interactions within these infections are therefore a major component of pathogenesis (9). These interactions can be both indirect, such as when one species manipulates the host immune system to the (dis)advantage of another species (10), or direct, such as when two or more species in close proximity vie for nutrients (11). An open question is to what extent polymicrobial interactions are antagonistic or mutualistic, as examples can be found for both (5, 12). An emerging paradigm from microbiome studies is that specific community compositions can increase host susceptibility to infection (13, 14). This phenomenon can be partly explained by synergistic interspecies interactions that enhance the virulence of pathogens (15). While synergistic interactions between microbes clearly impact the fitness of host-associated microbial communities, elucidating the mechanisms controlling synergy within these complex communities has been challenging. To tackle this problem, several research groups have taken advantage of simplified (reduced diversity) communities (16–18). These studies have shown that synergy can stem from both antagonistic (19) and mutualistic (20, 21) interactions.

We previously established a model bacterial community consisting of the opportunistic oral pathogen *Aggregatibacter actinomycetemcomitans* and the commensal oral bacterium *Streptococcus gordonii*. These bacteria coexist not only in the human oral cavity but also in infections of the heart and in abscesses of the skin, brain, and lung (22). *A. actinomycetemcomitans* and *S. gordonii* are both facultative anaerobes that can proliferate both in high- and low-oxygen environments, which has important consequences for their metabolisms. The two major modes of energy production are metabolic fermentation and respiration. As *S. gordonii* is an obligate fermenter, it only produces ATP by substrate-level phosphorylation, and therefore, its metabolism is largely unchanged in the presence of oxygen. An exception to this is the relative abundance at which *S. gordonii* produces its two major metabolites, l-lactate and hydrogen peroxide (H_2_O_2_), since H_2_O_2_ production by *S. gordonii* requires oxygen (23). In contrast, *A. actinomycetemcomitans* can both ferment and respire, where it can also gain energy from reducing an electron acceptor (such as oxygen) via an electron transport chain. This electron transport chain establishes a proton motive force that can be used to produce ATP by oxidative phosphorylation, and thus, respiration is generally a higher-ATP-yielding form of metabolism than fermentation.

A major discovery from our studies of the *A. actinomycetemcomitans-S. gordonii* community is that *S. gordonii* promotes the virulence of *A. actinomycetemcomitans*. This disease synergy depends on multiple factors, including metabolic cross-feeding and spatial organization. The metabolite l-lactate produced by *S. gordonii* is a preferred carbon source for *A. actinomycetemcomitans* (20), and catabolism of l-lactate in coinfection with *S. gordonii* is required for synergy in abscesses (21). In response to *S. gordonii*-produced H_2_O_2_, *A. actinomycetemcomitans* produces a biofilm-degrading enzyme that allows it to maintain a precise distance from *S. gordonii*, which is also required for synergy in abscesses (24). Key to both of these interactions is the presence of oxygen, since only in the presence of oxygen can *A. actinomycetemcomitans* consume l-lactate (21) and *S. gordonii* produce H_2_O_2_ (23). However, transcriptome sequencing (RNA-seq) examination of monoinfected *A. actinomycetemcomitans* abscesses revealed that *A. actinomycetemcomitans* metabolism *in vivo* is primarily anaerobic (25). These seemingly conflicting observations raise questions about the role of oxygen in *A. actinomycetemcomitans-S. gordonii* infections. Are oxygen levels constant but high enough to allow *A. actinomycetemcomitans* l-lactate catabolism/*S. gordonii* H_2_O_2_ production? Or alternatively, are oxygen levels dynamic, increasing in availability during coinfection?

In this study, we used an open-ended genome-wide approach, transposon sequencing (Tn-seq), to assess how *A. actinomycetemcomitans* physiology is altered by coinfection with *S. gordonii*. Using this approach, we can simultaneously test the fitness of thousands of mutants *en masse* (10, 11, 26). We decided on Tn-seq to investigate mutant fitness directly over another common genomic approach, RNA-seq, since previous studies by our group (27) and others (28) have found that transcriptomic data are poor predictors of mutant fitness. In addition, our goal was not to examine how *A. actinomycetemcomitans* responds to *S. gordonii* but, instead, to identify *bona fide* fitness determinants that *A. actinomycetemcomitans* needs to survive with *S. gordonii*. Knowing the importance of oxygen to *A. actinomycetemcomitans-S. gordonii* interactions, our strategy was to first use Tn-seq to expand our knowledge of oxygen-influenced processes in *A. actinomycetemcomitans* and then leverage these data to assess oxygen levels in the infection site.

## RESULTS

### Identifying signatures of anoxic and oxic growth in *A. actinomycetemcomitans*.

To perform Tn-seq in *A. actinomycetemcomitans*, we generated a pool of ~10,000 transposon mutants, corresponding to ~1 insertion per 200 bp along the 2.1-Mbp *A. actinomycetemcomitans* genome. As one of our goals was to understand the role of oxygen in *A. actinomycetemcomitans* pathogenesis, we first used Tn-seq to identify *A. actinomycetemcomitans* fitness determinants under defined oxygen levels *in vitro*. To do this, we compared the mutant pool after it was grown in the absence of oxygen to the pool after it was grown in the presence of oxygen. We used a complex medium for these experiments, since a minimal medium would deplete many of the mutants from the input pool and we wanted to maximize the number of genes we could assess that are affected by oxygen. It should be noted that, while powerful, Tn-seq does not provide information on all genes that possess transposon insertions due to cross-complementation and the low abundance of some mutants. Despite these limitations, we identified 214 genes and intergenic regions as fitness determinants for anoxic or oxic growth (see Table S1 in Text S2 in the supplemental material), of which oxic fitness determinants make up a larger proportion.

**FIG 1:**
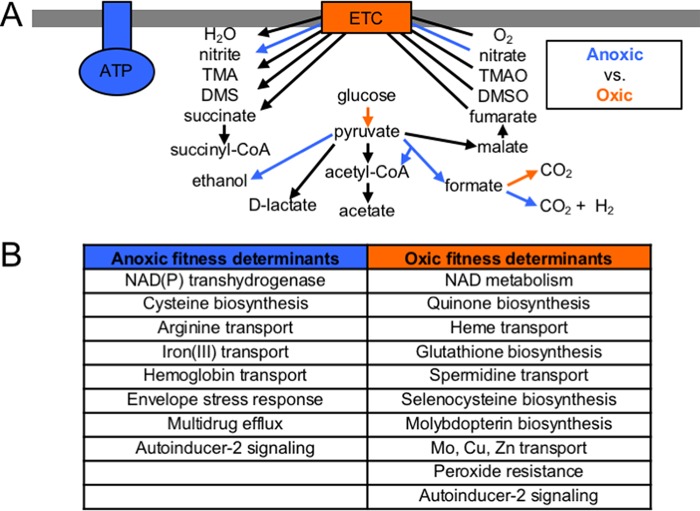
Metabolic pathways required for anoxic and oxic growth *in vitro*. (A) Blue and orange indicate pathways required for anoxic and oxic growth, respectively. Each arrow represents an enzyme(s). The box embedded in the thick gray bar (symbolizing the membrane) represents the electron transport chain (ETC). The structure labeled ATP represents ATP synthase. TMAO, trimethylamine *N*-oxide; TMA, trimethylamine; DMSO, dimethyl sulfoxide; DMS, dimethyl sulfide; CoA, coenzyme A. (B) Cellular processes required for anoxic and oxic growth. See Tables S1 and S2 in Dataset S1 in the supplemental material for a full summary.

To identify enriched functions in these fitness determinants, we used the Clusters of Orthologous Groups (COGs) classification system (29). We found that the most enriched COGs among anoxic fitness determinants are “Energy production” and “Signal transduction” (see Fig. S1A in the supplemental material). The enrichment of the COG Energy production is not surprising, since *A. actinomycetemcomitans* is known to use different modes of energy production, either fermentation or respiration, depending on the presence of oxygen (21). As many of the pathways determining whether *A. actinomycetemcomitans* is fermenting or respiring are in central metabolism, we reconstructed central metabolism *in vitro* (Fig. 1A) to help in later distinguishing anoxic and oxic environments *in vivo*. We saw that known anaerobic enzymes, such as pyruvate formate lyase and the hydrogenase-linked formate dehydrogenase complex, are important for anoxic growth, whereas a respiratory formate dehydrogenase is required for oxic growth. These findings agree with expectations based on previous metabolite profile measurements (21), where it was found that anaerobically, *A. actinomycetemcomitans* produces a mixture of mostly formate and acetate (via pyruvate formate lyase) and succinate, whereas aerobically, *A. actinomycetemcomitans* produces a mixture of mostly acetate and d-lactate. Global regulators of central metabolism, fumarate-nitrate reductase (FNR) and cyclic AMP receptor protein (CRP), are also required for anoxic growth, contributing to the enrichment of the COG Signal transduction among anoxic fitness determinants.

Based on fold changes in mutant abundance, the most important genes for anoxic growth encode components of ATP synthase (Fig. 1A). This is surprising, since ATP synthase is the primary producer of ATP in respiring cells, where it acts to couple the import of protons to the generation of ATP. However, *A. actinomycetemcomitans* can switch between fermentation and respiration, depending on the availability of electron acceptors, and therefore, as in other facultative anaerobes (30), ATP synthase can be disrupted. During fermentative growth in these bacteria, ATP synthase can also be used in reverse to break down ATP, acting to export protons from the cells. This activity can be beneficial for either establishing a proton gradient or resisting acid stress (31), either of which could be its role in *A. actinomycetemcomitans* during anoxic growth.

Many other cellular processes are also fitness determinants in the absence or presence of oxygen (Fig. 1B; see also Tables S1 and S2 in Dataset S1 in the supplemental material). Studying this list, we noted that the biosynthesis or transport of many cofactors is required for oxic growth, reflected in the enrichment of the COG Coenzyme metabolism among oxic fitness determinants (see Fig. S1A). These cofactors fulfill different needs during oxic growth, such as the electron transport chain (quinones and heme), oxidative stress resistance (glutathione and spermidine), and the respiratory formate dehydrogenase (selenocysteine and molybdopterin). From these data, we conclude that reconstruction of central metabolism from Tn-seq data can reveal biomarkers for *A. actinomycetemcomitans* aerobic/anaerobic growth.

### *A. actinomycetemcomitans* monoinfections contain both anoxic and oxic environments.

**FIG 2:**
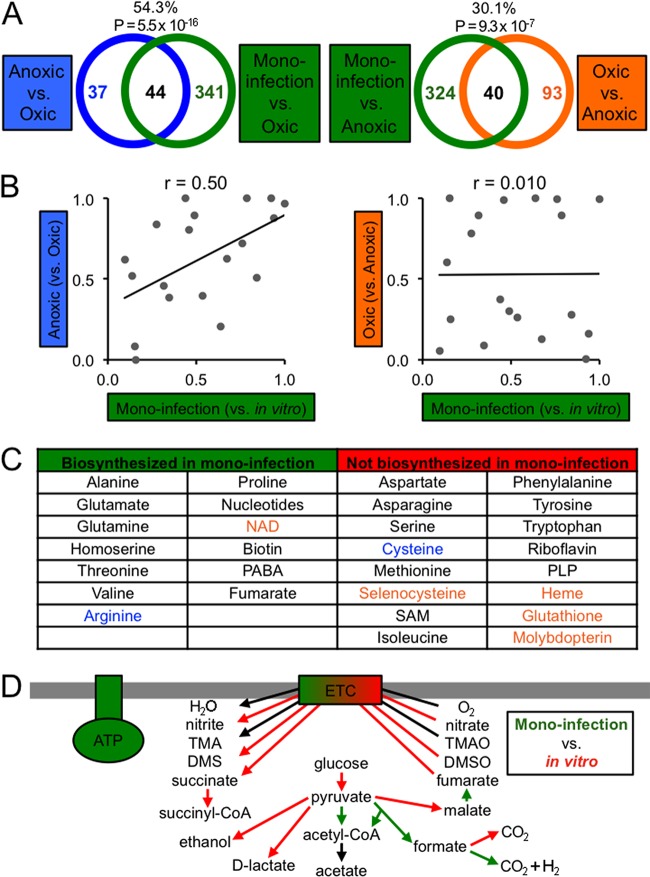
Anoxic growth is important for *A. actinomycetemcomitans* monoinfection. (A) Venn diagrams showing the overlap between monoinfection and anoxic (left) or oxic (right) growth *in vitro*. The percentages represent the percentage of anoxic (left) or oxic (right) fitness determinants that overlap monoinfection fitness determinants. The *P* values represent the significance of the enrichment of anoxic (left) or oxic (right) fitness determinants (one-tailed Fisher exact test). (B) Spearman’s rank correlation (*r*) between *P* values for the enrichment of COGs among monoinfection and anoxic (left) or oxic (right) fitness determinants (one-tailed Fisher exact test). Each point corresponds to an individual COG. (C) Biosynthetic requirements for monoinfection determined as described in the materials and methods. Metabolites in blue and orange are required for anoxic and oxic growth, respectively. PABA, *p*-aminobenzoic acid; SAM, *S*-adenosylmethionine; PLP, pyridoxal phosphate. See Table S5 in Dataset S1 in the supplemental material for a full summary. (D) Central metabolic pathways required for monoinfection. Green and red indicate pathways required for growth in the abscess and growth *in vitro*, respectively. Abbreviations not defined here are defined in the legend to Fig. 1 or in the text.

We next used a murine thigh abscess model to identify fitness determinants for the persistence of *A. actinomycetemcomitans in vivo*. We formed abscesses with the *A. actinomycetemcomitans* mutant pool and extracted DNA directly from these abscesses to perform Tn-seq. Comparing growth in the abscess to growth under both anoxic and oxic *in vitro* conditions, we found that over 18% of the genome is required for fitness in the abscess (see Table S1 in Text S2 in the supplemental material). To determine whether growth in the abscess is more like anoxic or oxic growth *in vitro*, we examined whether anoxic or oxic fitness determinants are enriched among monoinfection fitness determinants (Fig. 2A). Our results revealed that the percentage of anoxic fitness determinants overlapping monoinfection fitness determinants (54%) is higher than the percentage of oxic fitness determinants overlapping monoinfection fitness determinants (30%). However, both anoxic and oxic fitness determinants are significantly enriched among monoinfection fitness determinants (Fig. 2A), suggesting that the abscess has both anoxic and oxic zones.

We next looked for enriched functions among abscess fitness determinants, using the same COG classification system as before. We separately investigated enriched COGs among fitness determinants identified from the monoinfection versus oxic comparison and from the monoinfection versus anoxic comparison (see Fig. S1B in the supplemental material). We found that the two most significantly enriched COGs in the monoinfection versus oxic comparison, Energy production and Signal transduction, are also the most enriched COGs among anoxic fitness determinants, suggesting that persistence in the abscess is functionally similar to anoxic growth *in vitro*. To expand this analysis, we also examined the correlation in terms of the relative importance of all COGs, using the *P* values from the enrichment test (Fig. 2B). This analysis revealed that monoinfection COGs are more positively correlated with anoxic fitness determinants (Spearman’s *r* = 0.50) than with oxic fitness determinants (*r* = 0.010), supporting the hypothesis that the abscess is more anoxic than oxic, both in terms of overlapping genes and enriched functions.

After gaining this initial overview of enriched functions in monoinfection, we next focused on specific virulence factors, carbon sources, and transported substrates (see Fig. S2 and Table S3 in Dataset S1 in the supplemental material). We found that many virulence factors are required for *A. actinomycetemcomitans* to persist *in vivo*. Notable examples include leukotoxin, cytolethal distending toxin, and lipopolysaccharide metabolism, some of the most widely studied virulence factors in *A. actinomycetemcomitans* (32). Less-recognized *A. actinomycetemcomitans* virulence factors required in the abscess include type IV secretion and multiple toxin-antitoxin (TA) systems. Other virulence factors important in the abscess are related to attachment (e.g., tight adherence) (33), the biofilm lifestyle (e.g., the polysaccharide poly-GlcNAc), detoxification of reactive nitrogen species (e.g., nitroreductases), and the envelope stress response (e.g., the CpxAR two-component system).

Our genomic data set also allowed us to query the global biosynthetic requirements for *A. actinomycetemcomitans* in the abscess (Fig. 2C). As expected, many biosynthetic genes are required for *A. actinomycetemcomitans* to persist *in vivo*, indicating that several metabolites are unavailable in the infection site. However, in many cases, we found that the transporter(s) for a metabolite is instead required (see Fig. S2 in the supplemental material) and the biosynthetic genes are dispensable, indicating that the metabolite is available for import into *A. actinomycetemcomitans* (see Tables 5 and 6 in Dataset S1 for a full summary of this analysis). Transporters for many other substrates are also fitness determinants during monoinfection. These substrates include metals and reveal, for example, important iron sources for *A. actinomycetemcomitans* in monoinfection, such as ferric iron (see Fig. S2). Finally, we also assessed which requirements for *A. actinomycetemcomitans* to persist *in vivo* also occur *in vitro* under anoxic or oxic growth conditions. Most revealing from this analysis was that many of the biosynthetic/transport pathways in *A. actinomycetemcomitans* that are dispensable in monoinfection are required for oxic growth *in vitro*. Examples of this include selenocysteine biosynthesis (Fig. 1B and 2C) and spermidine transport (Fig. 1B; see also Fig. S2). These data indicate that anoxic conditions in the abscess alleviate the requirement for oxic growth-associated metabolites, again suggesting that the abscess is primarily anoxic.

Due to our success reconstructing central metabolism *in vitro* to gauge oxygen levels, we reconstructed central metabolism *in vivo* and found that, in general, pathways and global regulators that are fitness determinants for anoxic growth (Fig. 1B) are also fitness determinants in the abscess (Fig. 2D). Chief among these are pyruvate formate lyase, the anaerobic formate dehydrogenase, and the global regulators FNR and CRP. However, central metabolic pathways are not completely anaerobic, since pyruvate dehydrogenase, an aerobic complex, is required in the abscess, as well as a quinone biosynthetic gene that is also an oxic fitness determinant. These findings again support the notion that the abscess contains both anoxic and oxic properties.

Similar to anoxic growth *in vitro*, the most important fitness determinants in monoinfection are components of ATP synthase. We therefore decided to make a defined mutant deficient for ATP synthase through allelic replacement of the first gene in the ATP synthase operon, *atpB*. Using this mutant, we first confirmed its fitness defect under anoxic growth conditions by measuring final growth yields (see Fig. S3A in the supplemental material). As expected, the mutant reaches a significantly lower yield than the wild type. As discussed earlier, possible roles of ATP synthase during anoxic growth are to export protons in order to establish a proton gradient or to resist acid stress. We first explored the idea that ATP synthase is required during anoxic growth in order to establish a proton gradient. We reasoned that, if so, the provision of an electron acceptor should alleviate this requirement, since this would allow *A. actinomycetemcomitans* to export protons via its electron transport chain. As expected, providing either oxygen or one of the alternative electron acceptors trimethylamine *N*-oxide (TMAO) and dimethyl sulfoxide (DMSO) increases the growth yields of the ATP synthase mutant to the levels of the wild type grown under the same conditions (see Fig. S3A). We also explored the idea that ATP synthase is required during anoxic growth in order to resist acid stress. To support this idea, we first measured the final pHs of anoxic and oxic cultures, revealing that anoxic cultures reach a lower pH than oxic cultures (see Fig. S3B). This suggests that *A. actinomycetemcomitans* may experience greater acid stress during anoxic growth than during oxic growth. We then reasoned that buffering the pH would rescue the anoxic growth defect of the ATP synthase mutant. As expected, the mutant reaches significantly higher anoxic growth yields when the culture is buffered to pH 8 than when it is buffered to pH 6.4 (see Fig. S3C). However, though the yields of the mutant increase in buffered medium, they do not reach the same wild-type levels observed when provided an electron acceptor. Furthermore, the growth rates of the mutant are highest when it is supplied with oxygen (see Fig. S3D). These data indicate that, while ATP synthase can be important to *A. actinomycetemcomitans* for resisting acid stress, its primary role is likely establishing a proton gradient, since we observed that only respiratory conditions fully rescue the mutant’s growth defect.

### *S. gordonii* promotes *A. actinomycetemcomitans* respiratory metabolism *in vivo*.

**FIG 3:**
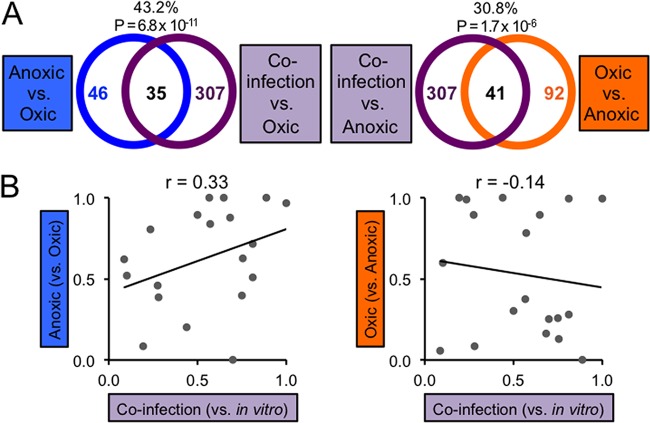
Coinfection stimulates *A. actinomycetemcomitans* oxic growth. (A) Venn diagrams showing the overlap between coinfection and anoxic (left) or oxic (right) growth *in vitro*. The percentages represent the percentage of anoxic (left) or oxic (right) fitness determinants that overlap coinfection fitness determinants. The *P* values represent the significance of the enrichment of anoxic (left) or oxic (right) fitness determinants (one-tailed Fisher exact test). (B) Spearman’s rank correlation (*r*) between *P* values for the enrichment of COGs among coinfection and anoxic (left) or oxic (right) fitness determinants (one-tailed Fisher exact test). Each point corresponds to an individual COG.

We next sought to examine how fitness determinants in *A. actinomycetemcomitans* are altered by the presence of *S. gordonii* in the abscess. *S. gordonii* is relevant because it is commonly found with *A. actinomycetemcomitans* in multispecies infections and is known to synergistically promote the virulence of *A. actinomycetemcomitans* in abscesses (21). We hypothesized that *S. gordonii* would increase the amount of oxygen in the abscess because the production of H_2_O_2_ requires oxygen and underlies many of the synergistic interactions of *S. gordonii* with *A. actinomycetemcomitans* (24). To test this hypothesis, we examined correlations and functional overlaps between Tn-seq results for coinfection and for the *in vitro* anoxic and oxic growth conditions. As with monoinfection, coinfection correlates more with the anoxic than the oxic *in vitro* condition (see Fig. S4A in the supplemental material), and also like monoinfection, it is significantly enriched for both anoxic and oxic fitness determinants (Fig. 3A). However, a smaller percentage of anoxic fitness determinants (43%) overlap with coinfection than with monoinfection fitness determinants (54%) (Fig. 2A), suggesting that coinfection is less anoxic than monoinfection. Supporting this notion, principal component analysis revealed that coinfection, compared to monoinfection, lies farther from the anoxic condition (see Fig. S4B), and furthermore, the fold changes of anoxic fitness determinants tends to be less for coinfection than for monoinfection (see Fig. S4C), suggesting that genes required for anoxic growth *in vitro* are not as important in coinfection as they are in monoinfection.

To further strengthen the idea that *S. gordonii* promotes aerobic growth *in vivo*, we also examined how coinfection functionally compares to the *in vitro* anoxic and oxic conditions, using the same analysis of enriched COGs. Our primary finding was that Energy production, an enriched COG among anoxic and monoinfection fitness determinants, is not enriched among coinfection fitness determinants (see Fig. S1C in the supplemental material). We then ranked all COGs in order of importance (based on *P* values from the enrichment test) and assessed their correlations (Fig. 3B). Like monoinfection, coinfection fitness determinants are more correlated with anoxic (*r* = 0.33) than with oxic fitness determinants (*r* = –0.14). The strength of the correlation of coinfection with anoxic fitness determinants, however, is less than that of monoinfection (*r* = 0.50) (Fig. 2B). Together, these data indicate that during coinfection, *S. gordonii* promotes *A. actinomycetemcomitans* aerobic physiology.

**FIG 4:**
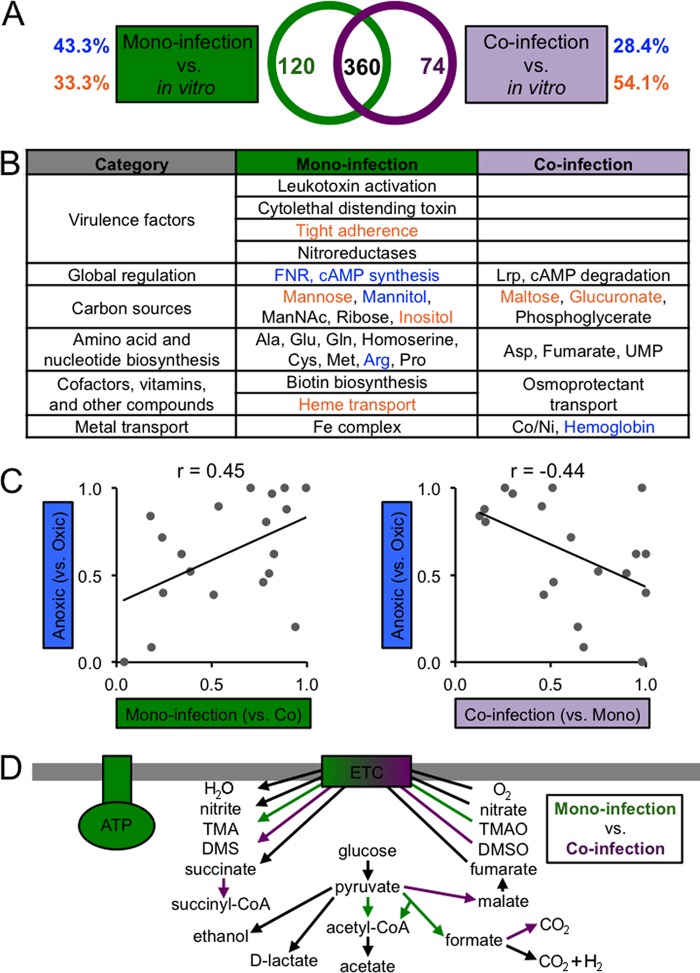
Fitness determinants specific to mono- and coinfection. (A) Venn diagram showing the numbers of fitness determinants specific to mono- and coinfection. Blue and orange indicate the percentages of fitness determinants associated with anoxic and oxic growth *in vitro*, respectively. (B) Cellular processes specific to mono- and coinfection. Processes in blue and orange are required for anoxic and oxic growth, respectively. cAMP, cyclic AMP. See Table S8 in Dataset S1 in the supplemental material for a full summary. (C) Spearman’s rank correlation (*r*) between *P* values for the enrichment of COGs among anoxic and monoinfection (left) or coinfection (right) fitness determinants (one-tailed Fisher exact test). Each point corresponds to an individual COG. (D) Central metabolic pathways required for mono- and coinfection. Green and purple indicate pathways required for mono- and coinfection, respectively. Abbreviations not defined here are defined in the legend to Fig. 1 or in the text.

Since the mono- and coinfection fitness determinants overlap by 360 genetic elements (Fig. 4A), we next set out to identify genetic elements that are specifically required in mono- or coinfection. We found that 120 genetic elements are specific to monoinfection and that 74 elements are specific to coinfection (Fig. 4A). More monoinfection-specific features are associated with anoxic growth (43%) than with oxic growth (33%). In contrast, more coinfection-specific features are associated with oxic growth (54%) than with anoxic growth (28%). This finding further supports our hypothesis that *S. gordonii* enhances oxygen levels in the abscess. Further analysis highlighted specific cellular processes that are unique to mono- or coinfection (Fig. 4B; see also Table S7 in Dataset S1 in the supplemental material). Notably, many virulence factors are required only in monoinfection. Furthermore, *A. actinomycetemcomitans* appears to use more carbon sources and to have greater biosynthetic requirements in mono- than in coinfection. Finally, central metabolic pathways and global regulators specific to monoinfection are primarily associated with anoxic growth, reinforcing the idea that *A. actinomycetemcomitans* is less exposed to oxygen in monoinfection than in coinfection with *S. gordonii*.

**FIG 5:**
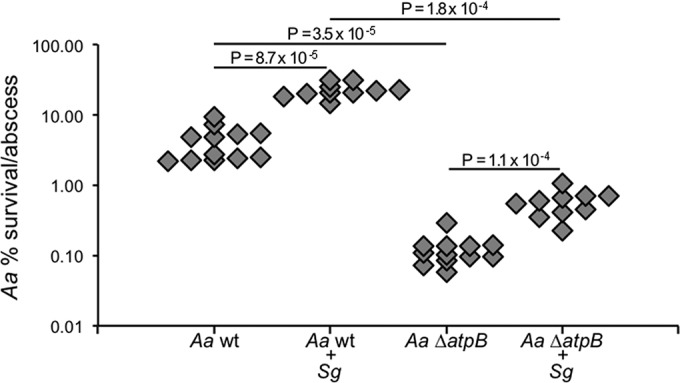
*S. gordonii* exhibits synergy with wild-type (wt) *A. actinomycetemcomitans* and the *A. actinomycetemcomitans ΔatpB* mutant. Abscesses formed with the indicated strains were harvested at 3 days postinfection, and CFU were determined. Each symbol represents a single abscess. Data represent the results for 2 biological replicates (*n* ≥ 10 mice). % survival/abscess (*y* axis) was calculated using the output and input CFU/abscess. Statistical significance was determined by a two-tailed Mann-Whitney *U* test.

As a further test of how coinfection affects oxygen levels *in vivo*, we also examined differential fitness when directly comparing mono- to coinfection. We hypothesized that many of the coinfection fitness determinants would be involved in adapting to oxic conditions. Overall, we found that *S. gordonii* exacerbates as much as it alleviates *A. actinomycetemcomitans* fitness determinants in the abscess; that is, the number of fitness determinants more required is approximately the same as that less required in coinfection compared to monoinfection (see Table S1 in Text S2 in the supplemental material). Further analysis of these fitness determinants revealed that the only significantly enriched COG, Energy production, is among the monoinfection-specific fitness determinants (see Fig. S1D). As this COG is also one of the most enriched COGs among the anoxic fitness determinants, it suggests that the major consequence to *A. actinomycetemcomitans* physiology in coinfection is a shift away from anaerobic metabolism. Additional support for this can be seen in the fact that the relative importance of all COGs is positively correlated between monoinfection and anoxic fitness determinants but negatively correlated between coinfection and anoxic fitness determinants (Fig. 4C). A reconstruction of central metabolism for mono- and coinfection fitness determinants revealed that anoxic enzymes/regulators, such as pyruvate formate lyase, FNR, CRP, and ATP synthase, are primarily required in monoinfection, whereas oxic enzymes/regulators, such as the aerobic formate dehydrogenase and a quinone biosynthetic gene, are primarily required in coinfection (Fig. 4D). To confirm this reconstruction, we coinfected the *A. actinomycetemcomitans* wild type and our ATP synthase mutant with *S. gordonii* and compared the survival in abscesses that were infected alone or with both species. As previously shown (21), *S. gordonii* increases wild-type *A. actinomycetemcomitans* survival, and as predicted by our Tn-seq data, *S. gordonii* also increases the survival of the ATP synthase mutant (Fig. 5). The survival of *S. gordonii* is not negatively affected by coinfection with the ATP synthase mutant compared to coinfection with *A. actinomycetemcomitans* wild type (see Fig. S6). As ATP synthase is required only under anoxic, fermentative conditions, our infection data show that *S. gordonii* generates oxic, respiratory conditions in the abscess that mitigate the growth defect of the ATP synthase mutant in monoinfected abscesses.

## DISCUSSION

*A. actinomycetemcomitans* is an opportunistic pathogen that rarely inhabits infection sites alone. Its ability to establish infections therefore likely evolved in the context of other bacterial species that may influence *A. actinomycetemcomitans* pathogenesis either by promoting or inhibiting its growth within the host. These interactions are probably diverse and occur simultaneously in a complex network, requiring high-throughput techniques to grasp their scope. In this study, we used sequencing-based mutant fitness profiling (Tn-seq) to globally assess *A. actinomycetemcomitans* requirements for abscess infection in monoculture and coculture with *S. gordonii*, a commensal species commonly found with *A. actinomycetemcomitans* in the oral cavity and abscesses.

Previous work showed that *S. gordonii* promotes the virulence of *A. actinomycetemcomitans* through multiple mechanisms. These include cross-feeding by *A. actinomycetemcomitans* on *S. gordonii*-produced l-lactate (20, 21) and spatial organization by *A. actinomycetemcomitans* in response to *S. gordonii*-produced H_2_O_2_ (24). Common to these mechanisms is a reliance on oxygen. This is because only aerobically can *A. actinomycetemcomitans* consume l-lactate and *S. gordonii* produce H_2_O_2_. An open question from this previous work was the status of oxygen between *A. actinomycetemcomitans* mono- and coinfections. Are the oxygen levels constant, or do they dynamically increase from mono- to coinfection? We hypothesized the latter, since previous RNA-seq data on *A. actinomycetemcomitans* in monoinfected abscesses suggested that *A. actinomycetemcomitans* primarily engages in anaerobic fermentation when it is by itself (25), but previous coinfection data suggested that, when it is with *S. gordonii*, *A. actinomycetemcomitans* primarily uses the strictly respiratory carbon source l-lactate (21). As it is not currently possible to precisely measure oxygen levels near infecting bacteria in abscesses, we set out to first use Tn-seq to increase our understanding of *A. actinomycetemcomitans* physiology in defined anoxic and oxic growth environments *in vitro* and then leverage these genomewide data to infer the oxygen levels in abscess infections.

Our data provide several lines of evidence that the abscess holds mixed oxygen levels, both anoxic and oxic, but that the presence of *S. gordonii* increases the oxygen levels, ultimately shifting *A. actinomycetemcomitans* metabolism from fermentation to respiration. Specifically, we saw that *A. actinomycetemcomitans* mutant profiles in the abscess overlap extensively with those from both anoxic and oxic growth *in vitro*, but functionally they are more similar to the anoxic condition. The presence of *S. gordonii*, however, stimulates a less anoxic mutant profile, a shift that is reflected by the dispensability of *A. actinomycetemcomitans* anaerobic pathways in coinfection. Most importantly, we found that ATP synthase, the strongest signature for *A. actinomycetemcomitans* anoxic growth, is required in monoinfection. However, in coinfection, the ATP synthase mutant exhibits the same ~5-fold increase in cell yield as the wild type, indicating that ATP synthase is not required for *A. actinomycetemcomitans* synergy with *S. gordonii* (Fig. 5). Experiments *in vitro* showed that the *A. actinomycetemcomitans* ATP synthase mutant reaches lower growth yields under anoxic, strictly fermentative conditions but that it could be rescued by each of the electron acceptors tested, including oxygen, TMAO, and DMSO (see Fig. S3A in the supplemental material). The parallelism between the synergistic effect of *S. gordonii* on *A. actinomycetemcomitans* in the abscess and the effect of electron acceptors on the ATP synthase mutant *in vitro* suggests that *S. gordonii* fulfills the role of providing *A. actinomycetemcomitans* with an electron acceptor to promote respiration *in vivo*. While it is also possible that *S. gordonii* could raise the pH in the abscess, we find this model unlikely, since the primary *S. gordonii* metabolite, l-lactate, would likely decrease the pH within the abscess, exacerbating the ATP synthase growth defect. While our data support the specific electron acceptor provided by *S. gordonii* being oxygen, it is also possible that it could be an alternative electron acceptor, such as TMAO or DMSO, which were shown *in vitro* to also rescue the ATP synthase mutant (see Fig. S3A). However, experiments with an *A. actinomycetemcomitans* TMAO/DMSO respiration mutant showed that it is as virulent as the wild type in coinfections with *S. gordonii* (see Fig. S6), indicating that *S. gordonii* likely does not provide TMAO/DMSO to *A. actinomycetemcomitans* in coinfections, further supporting the role of oxygen.

While it is clear that *S. gordonii* promotes *A. actinomycetemcomitans* aerobic respiration in the abscess, the mechanism by which this occurs is not known. One hypothesis is that, due to consumption of O_2_ by pyruvate oxidase (which produces H_2_O_2_), *S. gordonii* creates a more expansive and steeper O_2_ gradient that enhances diffusion into the regions surrounding the bacteria. As *A. actinomycetemcomitans* and *S. gordonii* are highly colocalized in the abscess (24) and *A. actinomycetemcomitans* can produce O_2_ from H_2_O_2_, this increased diffusion likely increases the levels of O_2_ for *A. actinomycetemcomitans* aerobic respiration. Supporting this hypothesis, *A. actinomycetemcomitans* mechanisms for detoxifying H_2_O_2_ are not only required for full virulence in coinfection (24) but are also known to be capable of stimulating respiration (quinol peroxidase) (34). A direct test of the importance of H_2_O_2_ production by *S. gordonii* is made challenging by the fact that a mutant deficient in pyruvate oxidase (*spxB*) does not persist in the abscess model (21). A second hypothesis is that the presence of *S. gordonii* enhances the inflammatory response in coculture infections that ultimately results in elevated H_2_O_2_ levels. These hypotheses are not mutually exclusive, and efforts are under way to understand the basis for the *A. actinomycetemcomitans* respiratory shift in coinfection.

It is also noteworthy that although the ATP synthase mutant benefits from respiratory conditions, it is by definition not capable of generating ATP from respiration. This suggests that the benefit the ATP synthase mutant gains from coculture with *S. gordonii* is independent of a higher level of ATP production. Instead, this benefit likely comes from the proton gradient that *A. actinomycetemcomitans* can create when respiring in the presence of *S. gordonii*. Creating a proton gradient is a role that in wild-type *A. actinomycetemcomitans* can be performed by ATP synthase when operating in reverse under strictly fermentative conditions. Our data therefore decouple these two functions of ATP synthase, revealing that the benefit gained from coculture with *S. gordonii* is not limited to producing more ATP under respiratory conditions but also derives from generating a proton gradient under fermentative conditions. We believe the benefits provided by these two functions are reflected by the difference in cell counts of the wild type and the ATP synthase mutant in our infection data (Fig. 5). Since the ATP synthase mutant cannot generate ATP from respiration, its higher growth yields from coinfection with *S. gordonii* must be due to the increased ability of *A. actinomycetemcomitans* to establish a proton gradient. However, the ATP synthase mutant is not fully rescued to wild-type cell numbers because it cannot receive the benefit of higher ATP production. The difference in survival between wild-type *A. actinomycetemcomitans* and the ATP synthase mutant is therefore strongly indicative that *A. actinomycetemcomitans* respires and gains more ATP when it is in coculture with *S. gordonii*. Based on these collective data, we propose a new paradigm, termed cross-respiration, for multispecies interactions. Similar to a cross-feeding interaction, in which one bacterium produces a metabolite that another bacterium can use as food, cross-respiration is where one bacterium provides respiratory electron acceptors that redirect metabolism in another bacterium from fermentation to respiration. While similar host-microbe interactions have been described in the gut (3), we distinguish cross-respiration as an interaction that occurs specifically between microbes. In the context of *A. actinomycetemcomitans-S. gordonii* infection, the *S. gordonii*-induced shift to a respiratory metabolism enhances not only the fitness of *A. actinomycetemcomitans* but also its pathogenesis. Although the studies described herein were limited to a model abscess infection, we suggest they also have relevance for the oral cavity, where a diversity of other *Streptococcus* species also produce H_2_O_2_ (35) and associate with *A. actinomycetemcomitans* (36, 37). Based on the prevalence with which microbes produce H_2_O_2_ and, potentially, other sources of respiratory electron acceptors, such as TMAO (38), we propose that cross-respiration is likely a common mechanism whereby commensal bacteria promote the virulence of pathogens.

## MATERIALS AND METHODS

### Strains, media, and growth conditions.

The strains, media, and growth conditions used for routine cultures and specific experiments are described in the materials and methods in Text S1 in the supplemental material.

### Construction of *A. actinomycetemcomitans* mutants.

The construction of the *A. actinomycetemcomitans* ATP synthase and TMAO/DMSO reductase mutants is described in the materials and methods in Text S1 in the supplemental material.

### Tn-seq.

Details on how the *A. actinomycetemcomitans* mutant pool was generated, how Illumina sequencing libraries were prepared, and how Tn-seq data were analyzed are provided in the materials and methods in Text S1 in the supplemental material.

### Abscess model.

The murine thigh abscess infection model is described in the materials and methods in Text S1 in the supplemental material. Animal studies were conducted in accordance with the National Institutes of Health Guide for the Care and Use of Laboratory Animals. Animal protocols were approved by the Institutional Animal Care and Use Committees of The University of Texas at Austin and Texas Tech University Health Sciences Center.

### Data accession numbers.

Tn-seq sequencing data are available at the National Center for Biotechnology Information Sequence Read Archive (NCBI SRA) under accession number SRP070130. PacBio sequencing data are available at the NCBI SRA under accession number SRP059980. The genome assembly and annotation data are available at GenBank under accession number CP012958.

## SUPPLEMENTAL MATERIAL

Text S1  Supplemental materials and methods, references, and figure legends. Download Text S1, DOCX file, 0.03 MB

Text S2  Supplemental tables, protocols, and scripts for materials and methods. Download Text S2, DOCX file, 0.7 MB

Figure S1  Enrichment of Clusters of Orthologous Groups (COGs) among fitness determinants. Download Figure S1, JPG file, 0.1 MB

Figure S2  Requirements for monoinfection. Download Figure S2, TIF file, 1.2 MB

Figure S3  The *A. actinomycetemcomitans* ATP synthase mutant is rescued by providing electron acceptors or buffering the pH. Download Figure S3, JPG file, 0.1 MB

Figure S4  Coinfection shifts *A. actinomycetemcomitans* away from anoxic growth. Download Figure S4, TIF file, 2.9 MB

Figure S5  Requirements for mono- and coinfection. Download Figure S5, TIF file, 2 MB

Figure S6  Virulence of *S. gordonii* and the *A. actinomycetemcomitans* TMAO/DMSO reductase mutant. Download Figure S6, JPG file, 0.1 MB

Dataset S1  Tables summarizing fitness determinants identified in Tn-seq experiments. Download Dataset S1, DOCX file, 0.2 MB

Dataset S2  Spreadsheets showing raw differential fitness analyses and summarizing fitness determinants identified in Tn-seq experiments. Download Dataset S2, XLSX file, 2.9 MB
