# Supplementary material for: A Commensal Bacterium Promotes Virulence of an Opportunistic Pathogen via Cross-Respiration
Source: mBio. 2016 Jun 28;7(3):e00782-16. doi: 10.1128/mBio.00782-16 (PMC4916382; doi:10.1128/mBio.00782-16)
Supplement: Text S1 — Supplemental materials and methods, references, and figure legends. Download [file mbo003162854s1.docx]

**Supplemental Material**

**Materials and Methods**

*Strains, media, and growth conditions.* Strains and plasmids are listed in Appendix Table 2. *Aa* strains VT1169 and 624 were used for all experiments. *Aa* was routinely grown in filter-sterilized (1) tryptic soy broth (BD Difco) supplemented with 0.5% yeast extract (Fluka) (TSBYE) or on autoclaved tryptic soy agar (BD Difco) supplemented with 0.5% yeast extract (Fluka) (TSAYE). *Aa* grown under oxic conditions was incubated in a 5% CO_2_ atmosphere at 37ºC with shaking at 250 rpm. *Aa* grown under anoxic conditions was incubated in an anaerobic chamber (Coy) in an 85% N_2_, 10% CO_2_, 5% H_2_ atmosphere at 37ºC with shaking at 250 rpm. *S. gordonii* was routinely grown in TSBYE in a 5% CO_2_ atmosphere at 37ºC without shaking. *E. coli* was routinely grown in LB at 37ºC with shaking at 250 rpm.

*Generation of the Aa mutant pool.* *Aa* VT1169 was mutagenized with mini-Tn10 by conjugation with *E. coli* largely as described (2). Conjugations were carried out for up to 24 hours or more, plated onto selective media, and incubated under oxic conditions for 3-6 days. Resulting colonies were pooled together with a cell spreader into TSBYE, aliquotted, and frozen. Aliquots from separate conjugations were combined, grown under oxic conditions in TSBYE with antibiotics, and aliquotted ~100x to generate the final mutant pool (available upon request).

*Selection experiment to identify anoxic and oxic fitness determinants.* An aliquot of the mutant pool was thawed, and ~100 µl was diluted to OD_600_ = 0.005 per 50 ml volume of TSBYE in a 250 ml flask. Cultures were grown with shaking at 250 rpm under either anoxic or oxic conditions and monitored for up to ~27 hours until they reached an OD_600_ of ~1-1.5 (after 7-8 doublings). Biological replicates were repeated on separate days.

*Construction of the Aa ATP synthase mutant.* An ATP synthase mutant (*ΔatpB*) was constructed in *Aa* VT1169 by replacing the subunit A gene, *atpB* (VT1169_1844), with *aphA* (encoding kanamycin resistance) by double homologous recombination. The knockout construct was made by fusing ~1 kb DNA fragments upstream and downstream of *atpB* to *aphA* by overlap extension PCR as previously described (3) (see Appendix Table 3 for primer sequences). The resulting ~3 kb fragment was gel extracted, ligated into pGEM-T Easy (Promega), and transformed into DH5α using the TSS method (4) and selecting on 40 µg/ml kanamycin. A purified plasmid was double-digested with NotI (to release the knockout construct) and XmnI (to cut the vector and distinguish it from the construct, as they were approximately the same size). The NotI-digested construct was gel extracted, ligated into NotI-digested/CIP-treated pMRKO (5), and transformed into *E. coli* S17-1 *λpir* using the TSS method (4) and selecting on 40 µg/ml kanamycin. The knockout vector (pMRKO-ATP) was conjugated into *Aa* from S17-1 *λpir* largely as described (2). *E. coli* was counter-selected with 50 µg/ml nalidixic acid, and *Aa* recombinants were selected with 40 µg/ml kanamycin. 48 colonies were screened for sensitivity to 50 µg/ml spectinomycin. Allelic replacement in a sensitive clone was confirmed by PCR with primers ATP-UP-F and ATP-UP-R (Appendix Table 3). Tn-seq data confirmed that several genes in the ATP synthase operon are required for fitness during anoxic growth (Appendix Table 4).

*Growth yield experiments.* The *Aa* VT1169 wild-type and ATP synthase mutant were grown under oxic conditions overnight (for 16-20 hours) in TSBYE with 25 µg/ml nalidixic acid and, for the mutant only, 20 µg/ml kanamycin. Overnights were diluted 1:100 into fresh media without antibiotics and grown under oxic or anoxic conditions for ~24 hours. Cultures were vigorously vortexed (to disperse clumps), and the OD_600_ of 1 ml of undiluted culture was measured to quantify growth yield. Media for electron acceptor experiments was filter-sterilized TSBYE ± 40 mM electron acceptor. TMAO and DMSO (Sigma) stock solutions were made up at 2.5 M in diH_2_O and TSBYE, respectively. Media for pH experiments was TSBYE buffered with 20 mM MOPS and adjusted to pH 6.4, 7, or 8 before being sterilized by autoclaving. MOPS-buffered TSBYE was used within a few days after autoclaving (because of increasing inhibition towards *Aa*).

*Generation time experiments.* The *Aa* VT1169 wild-type and ATP synthase mutant were grown under oxic conditions for 12 hours in TSBYE. Strains were diluted to an OD_600_ of 0.02-2 in a 6-7 ml volume of TSBYE, TSBYE + 40 mM TMAO or DMSO, or TSBYE pH 6.4, 7, or 8 (20 mM MOPS-buffered, filter-sterilized). Cultures were grown under oxic or anoxic conditions. Growth rate was monitored by measuring the OD_600_ of 0.7 ml aliquots of the cultures at 1.5 hour intervals.

*Construction of Aa TMAO/DMSO reductase mutants.* TMAO/DMSO reductase mutants were constructed in *Aa* strain 624 using the same strategy as described for the ATP synthase mutant, except that allelic replacement constructs were introduced into 624 via natural transformation rather than conjugation. Knockout constructs for single mutants were made by fusing ~1 kb DNA fragments upstream and downstream of a target locus to *aphA* (encoding kanamycin resistance) by overlap extension PCR as previously described (3). Target loci were *torYZ* (D7S_00103-00104) and *dmsABCD* (D7S_01379-01382) (see Appendix Table 3 for primer sequences, designed using the *Aa* strain D7S-1 genome sequence). Primers for building knockout constructs were designed to introduce the *Aa* uptake signal sequence (6) onto the 5’ and 3’ ends of constructs to promote uptake of the constructs by *Aa*. After overlap extension, knockout constructs were gel purified, and natural transformation of *Aa* with the purified constructs was performed largely as described (3, 6). The double mutant was constructed by fusing the up- and down-stream regions of *dmsABCD* to *aad9* (encoding spectinomycin resistance), instead of *aphA*, and naturally transforming the purified overlap extension product into the kanamycin-resistant *torYZ* single mutant. Antibiotic-resistant clones were confirmed by PCR with primers TorY-UP-F and TorZ-check-R for *torYZ* mutants and with primers DmsA-UP-F and DmsD-check-R for *dmsABCD* mutants.

*TMAO reduction experiments.* The *Aa* 624 wild-type or TMAO/DMSO reductase mutants were diluted into fresh TSBYE ± 50 mM TMAO in 1.5 ml microcentrifuge tubes and incubated statically in an anaerobic chamber for 2 days. Supernatants were collected, and the concentration of TMA (a readout for how much TMAO was reduced) was measured using a modified spectrophotometric method (7). An exact protocol is provided in the Appendix. Briefly, TMA was extracted from culture supernatants with toluene, picric acid was added to the extracts, and absorbance was measured at 410 nm on a plate reader. A standard curve made using known amounts of TMA was used to calculate TMA concentrations in the supernatants.

*Murine thigh abscess infection model.* The abscess model for Tn-seq experiments was performed as previously described (5). The mutant pool for these experiments was prepared as follows. 150 µl to 2 ml of the mutant pool was revived in TSBYE with spectinomycin, resuspended in fresh media with or without *Sg*, and injected in 100-200 µl volumes (representing 10^7^-10^8^ CFU) into each of 4 mice per biological replicate. After 3 days, abscesses were extracted and stored in RNAlater (Ambion).

The abscess model for experiments with the ATP synthase and TMAO/DMSO reductase mutants was performed as previously described (5) with minor modifications. 6 week old, female, Swiss Webster mice were ordered from Charles River and allowed to acclimate after shipment for at least 3 days. *Aa* and *Sg* were prepared for injection as follows. *Aa* strain VT1169 was grown under oxic conditions overnight (for 16-20 hours) in liquid TSBYE. *Sg* was grown without shaking overnight in liquid TSBYE. In contrast, *Aa* strain 624 (a strain with ‘rough’ colony morphology) was spread onto solid TSAYE and grown overnight into a lawn. This lawn was then scraped off the plate with a cell spreader and pooled into TSBYE, producing a suspension of cells that was much less clumpy than if it were grown in liquid. Cultures were gently washed, resuspended in fresh TSBYE, and adjusted to the appropriate density. *Aa* for mono-infections was adjusted to an OD_600_ = 0.3-0.4 (corresponding to ~1 x 10^7^ CFU/100 µl). *Aa* and *Sg* for co-infections were adjusted, respectively, to an OD_600_ = 0.6-0.8 (~2 x 10^7^ *Aa* CFU/100 µl) and OD_600_ = 0.4 (~2 x 10^7^ *Sg* CFU/100 µl), and prior to injection were mixed at a 1:1 ratio to give a final concentration of ~1 x 10^7^ CFU of each species per 100 µl.

Mice were prepared for injection as follows. Mice were anesthetized with isoflurane delivered by a precision vaporizer (Patterson Veterinary) that was set to flow oxygen at 1-2 L/min. Mice were initially induced with 5% isoflurane, and once anesthetized, they were maintained for the procedure with 1-2% isoflurane. The left inner thigh of each mouse was shaved, and Nair was massaged into the infection site for 1 minute to fully remove hair. The infection site was then disinfected with 70-75% ethanol. While the ethanol was allowed to fully dry, the cell suspension serving as the inoculum was gently mixed by pipetting and drawn into a syringe through a 30G needle. 100 µl of the inoculum was then subcutaneously injected into the left inner thigh, making sure to pull the needle out slowly to avoid leakage. After 3 days, mice were sacrificed by CO_2_ asphyxiation and cervical dislocation, and abscesses were harvested to count viable CFU. Each abscess was placed into a BeadBug tube pre-filled with 2.8 mm steel beads (Sigma) to which 900 µl TSBYE had been added, and homogenized for 30 seconds in a Mini-Beadbeater (Biospec). Homogenized abscesses were then serially diluted and plated onto TSAYE with 5 µg/ml vancomycin to count viable *Aa* CFU or TSAYE with 50 µg/ml streptomycin to count viable *Sg* CFU. Plates were incubated under oxic conditions for 2 days.

*Tn-seq sequencing libraries.* DNA from *in vitro* samples was extracted with the DNeasy Blood & Tissue kit (Qiagen). DNA from abscesses was extracted with phenol:chloroform largely as described (8, 9). Purified DNA was sheared in either a S220 Focused-ultrasonicator (Covaris) or Q880R Sonicator (Qsonica) until it reached a size distribution that centered between 0.3-1 kb.

Purified, sheared DNA was prepared for Illumina sequencing largely as described (2-PCR method) (9, 10). An exact protocol is provided in the Appendix. For *in vitro* samples, 2.5 µg of sheared DNA per sample was C-tailed in a 50 µl volume, purified with AMPure XP beads, and eluted in 25 µl H_2_O. For *in vivo* samples, 20 µg of sheared DNA per sample was C-tailed in a 400 µl volume, purified with a spin column, and eluted in 40 µl H_2_O. For *in vitro* samples, 1 PCR-1 reaction was set up per sample using 5 µl (250-500 ng) of C-tailed template, purified with AMPure XP beads, and eluted in 50 µl H_2_O. For *in vivo* samples, 4 PCR-1 reactions were set up per sample, each PCR using 10 µl (2.5-5 µg) of C-tailed template, purified with a single spin column per sample, and eluted in 50 µl H_2_O. The remainder of the protocol was identical for *in vitro* and *in vivo* samples (see Appendix).

Although not discussed in this manuscript, the initial *Aa* VT1169 mutant pool, prior to anoxic/oxic selection or abscess infection, was also sequenced and analyzed. DNA purified from mutant pool aliquots was prepared for Illumina sequencing largely as described (INSeq method) (8, 11). An exact protocol is provided in the Appendix.

As previously described (8), the transposon-specific primer (Tn10-2 in the 2-PCR protocol, INSeq-Tn10 in the INSeq protocol (see Appendix)) was designed to anneal close to the transposon’s 5’ and 3’ ends and direct DNA replication outwards into genomic DNA flanking the transposon. This design provided a source of quality control in downstream computational analyses. If a sequencing read was truly derived from the transposon, a known transposon sequence (23 bases long for 2-PCR libraries, 11 bases long for INSeq libraries) should appear in the read upstream of genomic DNA sequence.

Libraries were sequenced at the Genome Sequencing and Analysis Facility at the University of Texas at Austin on a HiSeq 2000 or 2500 (Illumina). 2-PCR libraries were sequenced on 1x100 single end runs, and INSeq libraries were sequenced on 2x100 paired end runs. Raw sequencing files were deposited into the NCBI Sequence Read Archive (accession no. SRP070130).

*Aa VT1169 genome sequencing, assembly, and annotation.* The genome of *Aa* strain VT1169 was sequenced to a depth of ~410x on a PacificBiosciences RS II sequencer using P5-P3 chemistry at the University of Michigan DNA sequencing core. Reads were assembled into a closed genome using the SMRT Analysis pipeline v2.3 (12). The genome was annotated with the NCBI Prokaryotic Genome Annotation Pipeline (PGAP) (13) and KEGG Automatic Annotation Server v2.0 (14). BRITE hierarchy annotations were obtained from the annotation of *Aa* strain ANH9381 (15) available on the KEGG website (<http://www.genome.jp/kegg-bin/show_organism?org=aao>) (16). Annotations were curated to reduce redundancies and inaccuracies. Raw PacBio sequences were deposited into the NCBI Sequence Read Archive (accession no. SRP059980). The genome assembly and curated annotation were deposited into Genbank under accession no. CP012958.

*Computational analyses.* Tn-seq data were analyzed largely as described (8). Scripts from (8), adapted for processing and mapping *Aa* Tn-seq libraries, are available in the Appendix. Analyses were performed on the Texas Advanced Computing Cluster and are described below.

As described above, Tn-seq libraries were prepared such that sequencing reads derived from the transposon should contain a known transposon sequence. Reads were therefore first filtered, using fqgrep v0.4.3 (<https://github.com/indraniel/fqgrep>), for those that contain the transposon sequence in its expected location within the read. Reads were then trimmed, using cutadapt v1.8.3 (<http://cutadapt.readthedocs.org/en/stable/index.html>), of 3’ low-quality bases and Illumina adapter sequences (for INSeq libraries) or poly-C-tails (for 2-PCR libraries). Reads less than 20 bases long after trimming were also removed (to improve mapping efficiency). Reads were then mapped to the VT1169 genome using bowtie2 v2.1.0 (17) with default -end-to-end parameters (requiring the entire read to map to the genome). Reads mapping with high quality and only to one location (MAPQ > 39) were then used to determine unique insertion sites, and the read count at each of these sites was tallied. Importantly, in determining insertion site locations, the 9 bp duplication associated with Tn10 insertion events (18) was accounted for, dramatically reducing the number of unique insertions identified. In examining the distribution of insertion site counts, it was observed that many sites with low counts lie adjacent to sites with high counts. As previously noted (19), this can arise from DNA polymerase slipping during DNA replication. To correct for this, the read counts of adjacent sites were collapsed onto the site with the highest count (the local maximum) using a custom script (see Appendix). Correcting for slippage removed up to 1,175 sites per sample (see Appendix Tables 5 and 6). As previously noted (20), multifork replication can inflate read counts for insertions close to the origin of replication. To correct for this, local smoothing (LOESS) was used as previously described (8, 20). At most, insertions close to the origin (the maximum value in the LOESS regression) were found to be inflated 2.3 fold relative to the terminus (the minimum value in the LOESS regression) (see Appendix Table 7). Insertion site locations were then mapped to genetic elements (genes and intergenic regions) using the intersect function in the BEDTools suite v2.25.0 (21). Insertion sites were mapped to both full-length features and features where the 3’ 10% of genes were trimmed (to exclude analysis of insertions that may not disrupt gene function). Reference files for mapping to these features were built using Microsoft Excel. The read count per feature, excluding counts contributed by sites mapping to multiple features, was then tallied using the sumif function in Microsoft Excel. Finally, differential fitness was calculated for LOESS-corrected sites, full-length genetic elements, and 3’ 10% trimmed genetic elements using DESeq2 v1.10.0 (22) with default parameters, except for incorporating normalization factors to remove biases related to gene length, which were calculated using EDASeq v2.4.0 (23) (see Fig. 1 in the Appendix for a plot showing how these normalization factors remove this bias). Features were considered differentially fit if they met one of the following criteria: (1) the adjusted p value for the full-length feature was less than 0.05, (2) the adjusted p value for the 3’ 10% trimmed feature was less than 0.05, or (3) at least 1 site was present with an adjusted p value less than 0.05, and the fold change of the site(s) had the same sign as the full-length feature (and the trimmed feature, when considering non-intergenic regions). The only exception was that in the mono- vs. co-infection comparison, non-adjusted p values were used for criteria (1) and (2). DESeq2 results files can be found in Dataset S2. Further analyses of these files, to identify important virulence factors, carbon sources, and biosynthetic/transport requirements, can also be found in Dataset S2. Summaries of these analyses can be found in Dataset S1.

*Determining Aa biosynthetic requirements in vivo.* A metabolite was considered not available, or biosynthesized *in vivo*, if at least 1 biosynthetic gene for the metabolite was required *in vivo*. A metabolite was considered available, or not biosynthesized *in vivo*, if all differentially fit biosynthetic genes for the metabolite were required only *in vitro*. Full summaries of this analysis for mono- and co-infection can be found in Tables 5 and 9 in Dataset S1. Many metabolites could not be assessed for availability because the known biosynthetic genes were not present in the *Aa* genome (indicating auxotrophy), or because mutants in those genes were not present in the mutant pool (indicating essentiality). A list of these metabolites is provided in Table 4 in Dataset S1.

**References**

1. Bhattacharjee MK, Sugawara K, & Ayandeji OT (2009) Microwave sterilization of growth medium alleviates inhibition of *Aggregatibacter actinomycetemcomitans* by Maillard reaction products. *J Microbiol Methods* 78(2):227-230.

2. Mintz KP (2004) Identification of an extracellular matrix protein adhesin, EmaA, which mediates the adhesion of *Actinobacillus actinomycetemcomitans* to collagen. *Microbiol* 150(8):2677-2688.

3. Stacy A*, et al.* (2014) Bacterial fight-and-flight responses enhance virulence in a polymicrobial infection. *Proc Natl Acad Sci U S A* 111(21):7819-7824.

4. Chung CT, Niemela SL, & Miller RH (1989) One-step preparation of competent *Escherichia coli*: transformation and storage of bacterial cells in the same solution. *Proc Natl Acad Sci U S A* 86(7):2172-2175.

5. Ramsey MM, Rumbaugh KP, & Whiteley M (2011) Metabolite cross-feeding enhances virulence in a model polymicrobial infection. *PLoS Pathog* 7(3):e1002012.

6. Wang Y, Goodman SD, Redfield RJ, & Chen C (2002) Natural transformation and DNA uptake signal sequences in *Actinobacillus actinomycetemcomitans*. *J Bacteriol* 184(13):3442-3449.

7. Anderson WG & Allen PJ (2011) An alternative approach for the measurement of trimethylamine oxide in body fluid samples of elasmobranchs. *J Fish Biol* 78(2):667-672.

8. Turner KH, Everett J, Trivedi U, Rumbaugh KP, & Whiteley M (2014) Requirements for *Pseudomonas aeruginosa* acute burn and chronic surgical wound infection. *PLoS Genet* 10(7):e1004518.

9. Turner KH, Wessel AK, Palmer GC, Murray JL, & Whiteley M (2015) Essential genome of *Pseudomonas aeruginosa* in cystic fibrosis sputum. *Proc Natl Acad Sci U S A* 112(13):4110-4115.

10. Klein BA*, et al.* (2012) Identification of essential genes of the periodontal pathogen *Porphyromonas gingivalis*. *BMC Genomics* 13:578.

11. Goodman AL, Wu M, & Gordon JI (2011) Identifying microbial fitness determinants by insertion sequencing using genome-wide transposon mutant libraries. *Nat Protoc* 6(12):1969-1980.

12. Chin CS*, et al.* (2013) Nonhybrid, finished microbial genome assemblies from long-read SMRT sequencing data. *Nat Methods* 10(6):563-569.

13. Angiuoli SV*, et al.* (2008) Toward an online repository of Standard Operating Procedures (SOPs) for (meta)genomic annotation. *OMICS* 12(2):137-141.

14. Moriya Y, Itoh M, Okuda S, Yoshizawa AC, & Kanehisa M (2007) KAAS: an automatic genome annotation and pathway reconstruction server. *Nucleic Acids Res* 35(Web Server issue):W182-185.

15. Chen C, Kittichotirat W, Chen W, Downey JS, & Bumgarner R (2012) Genome sequence of a serotype b non-JP2 *Aggregatibacter actinomycetemcomitans* strain, ANH9381, from a periodontally healthy individual. *J Bacteriol* 194(7):1837.

16. Kanehisa M, Sato Y, Kawashima M, Furumichi M, & Tanabe M (2016) KEGG as a reference resource for gene and protein annotation. *Nucleic Acids Res* 44(D1):D457-462.

17. Langmead B & Salzberg SL (2012) Fast gapped-read alignment with Bowtie 2. *Nat Methods* 9(4):357-359.

18. Halling SM & Kleckner N (1982) A symmetrical six-base-pair target site sequence determines Tn10 insertion specificity. *Cell* 28(1):155-163.

19. Gallagher LA, Shendure J, & Manoil C (2011) Genome-scale identification of resistance functions in *Pseudomonas aeruginosa* using Tn-seq. *mBio* 2(1):e00315-00310.

20. Zomer A, Burghout P, Bootsma HJ, Hermans PW, & van Hijum SA (2012) ESSENTIALS: software for rapid analysis of high throughput transposon insertion sequencing data. *PLoS one* 7(8):e43012.

21. Quinlan AR & Hall IM (2010) BEDTools: a flexible suite of utilities for comparing genomic features. *Bioinformatics* 26(6):841-842.

22. Love MI, Huber W, & Anders S (2014) Moderated estimation of fold change and dispersion for RNA-seq data with DESeq2. *Genome Biol* 15(12):550.

23. Risso D, Schwartz K, Sherlock G, & Dudoit S (2011) GC-content normalization for RNA-Seq data. *BMC Bioinformatics* 12:480.

**Figure Legends**

**Fig. S1. Enrichment of Clusters of Orthologous Groups (COGs) among fitness determinants.** Fold enrichment (y-axis) is defined as the percent of fitness determinants made up by a COG divided by the percent of the genome (excluding potentially essential genes) made up by the same COG. †, P < 0.1; *, P < 0.01 (one-tailed Fisher’s exact test). (A) COG enrichment analyses were performed on fitness determinants identified from (A) the anoxic vs. oxic comparison, (B) the mono-infection vs. oxic and mono-infection vs. anoxic comparisons, (C) the co-infection vs. oxic and co-infection vs. anoxic comparisons, and (D) the mono-infection vs. co-infection comparison.

**Fig. S2. Requirements for mono-infection.** Names in blue and orange are required for anoxic and oxic growth *in vitro*, respectively. A full summary can be found in Tables 3-6 in Dataset S1.

**Fig. S3. The *Aa* ATP synthase mutant is rescued by providing electron acceptors or buffering the pH.** (A) Growth yields (OD_600_) of the *Aa* strain VT1169 wild-type (wt) and ATP synthase mutant (*ΔatpB*) under fermentative and respiratory conditions. TMAO (trimethylamine N-oxide) and DMSO (dimethyl sulfoxide) were provided at 40 mM. Error bars, SD (n ≥ 3). *, P < 0.001 (two-tailed Student’s t test, comparing to the anoxic *ΔatpB* growth yield). (B) Final pH of oxic and anoxic *Aa* VT1169 cultures compared to a blank (no cells) control. Error bars, SD (n = 3). *, P < 0.001 (two-tailed Student’s t test). (C) Growth yields (OD_600_) of *Aa* VT1169 wt and *ΔatpB* under anoxic conditions in media buffered (with 20 mM MOPS) to pH 8, 7, or 6.4. Error bars, SD (n ≥ 3). *, P < 0.01; †, P < 0.1 (two-tailed Student’s t test, comparing to the pH 6.4 *ΔatpB* growth yield). (D) Generations times (in minutes) of *Aa* VT1169 wt and *ΔatpB*. Growth conditions are indicated. -O_2_, anoxic. +O_2_, oxic. TMAO, 40 mM trimethylamine N-oxide. DMSO, 40 mM dimethyl sulfoxide. 6.4-8, pH 6.4-8 (20 mM MOPS buffer). ±, SD (n ≥ 3).

**Fig. S4. Co-infection shifts *Aa* away from anoxic growth.** (A) ‘Genome’ indicates all genetic elements were considered in calculating Spearman’s rank correlation coefficients. ‘Anoxic vs. Oxic Fitness Determinants’ indicates only elements differentially fit in the anoxic vs. oxic comparison were considered. Bar heights represent the average of 4 pairwise comparisons between biological replicates (n = 2, per condition). Error bars, SD. *, P < 0.05 (two-tailed Student’s t test). (B) Principal Component Analysis (PCA) was conducted with the normalized read counts for only genetic elements differentially fit with >2 fold change between the anoxic vs. oxic and mono- vs. co-infection comparisons. Percentages indicate the variation captured by PC 1 (x axis) and 2 (y axis). (C) The x- and y-axes correspond to the fold change (log_2_) of fitness determinants in mono- and co-infection, respectively. Each point corresponds to a fitness determinant for anoxic growth (vs. oxic growth) that was also significant in mono-infection (vs. oxic growth) and/or co-infection (vs. oxic growth). Errors bars correspond to the standard error (log_2_). The dashed line corresponds to a slope of 1 (corresponding to equal fold change for mono- and co-infection). Points below the line therefore have a higher fold change in mono- than co-infection.

**Fig. S5. Requirements for mono- and co-infection.** Names in blue and orange are required for anoxic and oxic growth *in vitro*, respectively. A full summary can be found in Tables 8-10 in Dataset S1.

**Fig. S6. Virulence of *Sg* and the *Aa* TMAO/DMSO reductase mutant.** (A) Abscesses formed with the indicated strains were harvested at 3 days post-infection, and colony forming units (CFU) were determined. Each symbol represents a single abscess. Data represent 2 biological replicates (n ≥ 10 mice). % survival/abscess (y-axis) was calculated using the output and input CFU/abscess. Statistical significance was determined by a two-tailed Mann-Whitney U test. (B) TMAO reduction by the *Aa* strain 624 wild-type (wt), TMAO (*ΔtorYZ*), DMSO (*ΔdmsABCD*), and TMAO/DMSO reductase double mutant (*ΔtorYZ ΔdmsABCD*), determined as described in SI Materials and Methods. Error bars, SD (n = 2 technical replicates). (C) Virulence of the *Aa* TMAO/DMSO reductase mutant was assessed as described in (A). Data represent 2 biological replicates (n ≥ 5 mice).
