# Supplementary material for: A Commensal Bacterium Promotes Virulence of an Opportunistic Pathogen via Cross-Respiration
Source: mBio. 2016 Jun 28;7(3):e00782-16. doi: 10.1128/mBio.00782-16 (PMC4916382; doi:10.1128/mBio.00782-16)
Supplement: Text S2 — Supplemental tables, protocols, and scripts for materials and methods. Download [file mbo003162854s2.docx]

| Condition | Fitness determinants | % genome |
| --- | --- | --- |
| Anoxic (vs. Oxic) | 81 (70/11) | 3.8 |
| Oxic (vs. Anoxic) | 133 (119/14) | 6.3 |
| Mono-infection (vs. *in vitro*) | 480 (312/168) | 18.6 |
| Co-infection (vs. *in vitro*) | 434 (267/167) | 16.6 |
| Mono-inf (vs. Co-inf) | 173 (128/45) | 6.3 |
| Co-inf (vs. Mono-inf) | 157 (107/50) | 6.0 |

**Table 1. Summary of *Aa* fitness determinants identified by Tn-seq.** The numbers in the fitness determinants column (middle) follow the format: number of genetic elements (number of genes/number of intergenic regions). For example, ’81 (70/11)’ indicates that 81 genetic elements, of which 70 were genes and 11 were intergenic regions, were fitness determinants in the anoxic condition.

| Strain or plasmid | Description | Source |
| --- | --- | --- |
| *A. actinomycetemcomitans* VT1169 | wild-type, nalidixic acid^R^, rifampicin^R^, smooth colony morphology | (1) |
| *A. actinomycetemcomitans* VT1169 *ΔatpB* | ATP synthase mutant, kanamycin^R^ | This study |
| *A. actinomycetemcomitans* 624 | wild-type, naturally competent, rough colony morphology |  |
| *A. actinomycetemcomitans* 624 *ΔtorYZ* | TMAO reductase mutant, kanamycin^R^ | This study |
| *A. actinomycetemcomitans* 624 *ΔdmsABCD* | DMSO reductase mutant, kanamycin^R^ | This study |
| *A. actinomycetemcomitans* 624  *ΔtorYZ ΔdmsABCD* | TMAO/DMSO reductase double mutant, kanamycin^R^, spectinomycin^R^ | This study |
| *E. coli* DH5α | cloning strain |  |
| *E. coli* S17-1 *λpir* | conjugative strain |  |
| *S. gordonii* Challis DL1.1 | wild-type, streptomycin^R^ | ATCC 49818 |
| pVT1542 | mini-Tn10 mutagenesis vector, spectinomycin^R^ | (2) |
| pYGK | source of *aphA* | (3) |
| pGEM-T Easy | TA cloning vector, ampicillin^R^ | Promega |
| pMRKO | *Aa* suicide vector, spectinomycin^R^, source of *aad9* | (4) |
| pMRKO-ATP | *Aa* suicide vector for deleting *atpB* | This study |

**Table 2. Strains and plasmids used in this study.**

| Target or  Library prep | Name | Sequence (5'->3') | Underlined region |
| --- | --- | --- | --- |
| ATPase  upstream  fragment | ATP-UP-F | GAAGATCTAAGTGCGGTGCCTATAATTTAACCTCCGTGC | BglII |
|  | ATP-UP-R | GTCATTGGATGATTCAGCGTATAATTAAGACCTATTCAGCAGAAATGG | overlap extension (*aphA*) |
| ATPase downstream fragment | ATP-DN-F | GGTATGAGTCAGCAACACCTTCAATTTTTTATTAACCGGCTCTAGG | overlap extension (*aphA*) |
|  | ATP-DN-R | GAAGATCTAAGTGCGGTTTGAATACTGCCGGAAGG | BglII |
| *aphA* (kanamycin resistance gene) | Kan-F | TTATACGCTGAATCATCCAATGAC |  |
|  | Kan-R | GAAGGTGTTGCTGACTCATACC |  |
| *torYZ* upstream fragment | TorY-UP-F | AAGTGCGGTCGAGCTGCCGTAACG |  |
|  | TorY-UP-R | GTCATTGGATGATTCAGCGTATAATCAGGCAAAGTGCGG | overlap extension (*aphA*) |
| *torYZ* downstream fragment | TorZ-DN-F | GGTATGAGTCAGCAACACCTTCCGTATAAAAAAACAACCGCAC | overlap extension (*aphA*) |
|  | TorZ-DN-R | AAGTGCGGTGCAAGATGTTAAGATGAAAAGC |  |
| check primer | TorZ-check-R | TCATATGTCTATCTTCGCAAG |  |
| *dmsABCD* upstream fragment | DmsA-UP-F | AAGTGCGGTCATGCGTCGCAAAGG |  |
|  | DmsA-UP-R | GTCATTGGATGATTCAGCGTATAATGCATACGTAACGGACAG | overlap extension (*aphA*) |
| *dmsABCD* downstream fragment | DmsD-DN-F | GGTATGAGTCAGCAACACCTTCCGTTCGTTTTTATCGTTAACG | overlap extension (*aphA*) |
|  | DmsD-DN-R | AAGTGCGGTGCCGTAAGTGGTTTTGC |  |
| check primer | DmsD-check-R | TCGTTCTGATGGAACACG |  |
| *aad9* (spectinomycin resistance gene) | Spc-F-Kan | TTATACGCTGAATCATCCAATGACCGATTTTCGTTCGTGAATACATG | overlap extension (*aphA*) |
|  | Spc-R-Kan | GAAGGTGTTGCTGACTCATACCCATATGCAAGGGTTTATTGTTTTC | overlap extension (*aphA*) |
| INSeq | INSeq-Tn10 | biotin-GGCCGCGATTTTTACCAAAATCATTAGG | NotI |
| INSeq | Illumina primer 1 (P5) | AATGATACGGCGACCACCGAGA |  |
| INSeq | Illumina primer 2 (P7) | ATCTCGTATGCCGTCTTCTGCTTG |  |
| 2-PCR | Tn10-1 | biotin-TTTACACTGATGAATGTTCCGTTGCGCTGC |  |
| 2-PCR | olj376 | GTGACTGGAGTTCAGACGTGTGCTCTTCCGATCTGGGGGGGGGGGGGGGG |  |
| 2-PCR | Tn10-2 | AATGATACGGCGACCACCGAGATCTACACTCTTTCCCTACACGACGCTCTTCCGATCTNNNNNAGATGTGTATCCACCTTAACTTAATGATTTTTACC | random bases |
| 2-PCR | BC-- | CAAGCAGAAGACGGCATACGAGATXXXXXXGTGACTGGAGTTCAGACGTGTG | Illumina barcode |

**Table 3. Primers used in this study.** Highlighted regions correspond to the *Aa* uptake signal sequence for natural transformation (5).

| locus tag | name | avg count | strand | log_2_FC | padj | sites |
| --- | --- | --- | --- | --- | --- | --- |
| VT1169_1846 | *gidB* | 344 | - | -0.5 | 0.803 | 0 |
| VT1169_1845 | *atpI* | 27 | - | -2.1 | 1.000 | 0 |
| VT1169_1844 | *atpB* | 1084 | - | -2.4 | 0.000 | 2 |
| VT1169_1843 | *atpE* | 115 | - | -1.1 | 0.577 | 0 |
| VT1169_1842 | *atpF* | 7139 | - | -1.6 | 0.000 | 1 |
| VT1169_1841 | *atpH* | 50 | - | -5.3 | 1.000 | 1 |
| VT1169_1840 | *atpA* | 1938 | - | -1.9 | 0.000 | 5 |
| VT1169_1839 | *atpG* | 962 | - | -0.9 | 0.095 | 0 |
| VT1169_1838 | *atpD* | 3060 | - | -2.9 | 0.000 | 2 |
| VT1169_1837 | *atpC* | 11 | - | 0.3 | 1.000 | 0 |
| VT1169_1836 | *potD* | 13835 | + | 0.2 | 0.828 | 0 |

**Table 4. Transposon insertions in the ATP synthase locus.** Data shown are from the anoxic vs. oxic comparison. Average (avg) count is mean read count (sequencing depth). Log_2_ fold change (FC) is negative for genes impaired for anoxic growth. Adjusted *p* value (padj) is the *p* value corrected for multiple testing. Orange, ATP synthase genes. Gray, up- or down-stream genes. Red, log_2_FC < -1 or padj < 0.05 or >0 insertion sites with padj < 0.05.

| Condition | Replicate | Library prep | Total reads | Reads containing the Tn (%)^†^ | Reads after processing (%)^†^ | Reads mapping to *Aa* (%)^†^ | Total insertions identified | Insertions after correcting slippage |
| --- | --- | --- | --- | --- | --- | --- | --- | --- |
| Input* | 1 | INSeq | 3,227,246^#^ | 3,007,840 (93.2) | 2,923,261 (97.2) | 2,236,064 (76.5) | 9,819 | 8,644 |
| Anoxic | 1 | 2-PCR | 16,480,451 | 16,044,302 (97.4) | 15,483,223 (96.5) | 14,614,798 (94.4) | 11,839 | 11,016 |
|  | 2 |  | 16,192,551 | 15,843,294 (97.8) | 15,396,663 (97.2) | 14,507,760 (94.2) | 10,987 | 10,161 |
| Oxic | 1 |  | 18,125,356 | 17,602,993 (97.1) | 17,033,391 (96.8) | 16,072,395 (94.4) | 10,568 | 9,720 |
|  | 2 |  | 17,835,468 | 17,350,457 (97.3) | 16,773,686 (96.7) | 15,855,074 (94.5) | 11,675 | 10,789 |
| Mono-infection | 1 |  | 53,360,274 | 35,536,166 (66.6) | 18,105,657 (50.9) | 15,535,124 (85.8) | 8,662 | 7,969 |
|  | 2 |  | 44,834,420 | 34,634,582 (77.2) | 20,745,381 (59.9) | 18,593,809 (89.6) | 16,366 | 15,273 |
| Co-infection | 1 |  | 53,431,462 | 43,070,778 (80.6) | 27,341,608 (63.5) | 23,011,272 (84.2) | 12,368 | 11,369 |
|  | 2 |  | 45,767,739 | 39,389,554 (86.1) | 28,537,459 (72.4) | 24,640,277 (86.3) | 13,987 | 12,922 |

**Table 5. Summary of Tn-seq analysis.** ‘Reads containing the Tn’ is how many reads contained the transposon sequence in its expected location within each read. ‘Reads after processing’ is how many reads remained after trimming 3’ low-quality bases, trimming 3’ non-genomic DNA sequences, and removing reads less than 20 bases long. ‘Reads mapping to *Aa*’ is how many reads mapped to the *Aa* genome with high quality. ‘Insertions identified’ is how many unique insertions were identified after accounting for the 9 bp duplication associated with Tn10 insertion events. ‘Insertions after correcting slippage’ is how many insertions remained after correcting for polymerase slippage.

*Input is an aliquot of the *Aa* VT1169 mutant pool, prior to any growth conditions.

^#^The original fastq file for the INSeq sample was corrupted, so ‘total reads’ for this condition is how many raw reads contained the transposon sequence.

^†^Percentages are relative to the value in the adjacent left column.

| Condition | Replicate | Total sites (with ambiguous sites) | Sites remaining after removing ambiguous sites | Sites remaining after correcting for slippage | Total sites removed |
| --- | --- | --- | --- | --- | --- |
| Input | 1 | 9,819 | 9,640 | 8,644 | 1,175 |
| Anoxic | 1 | 11,839 | 11,839 | 11,016 | 823 |
|  | 2 | 10,987 | 10,987 | 10,161 | 826 |
| Oxic | 1 | 10,568 | 10,568 | 9,720 | 848 |
|  | 2 | 11,675 | 11,671 | 10,789 | 886 |
| Mono-infection | 1 | 8,662 | 8,662 | 7,969 | 693 |
|  | 2 | 16,366 | 16,359 | 15,273 | 1,093 |
| Co-infection | 1 | 12,368 | 12,366 | 11,369 | 999 |
|  | 2 | 13,987 | 13,985 | 12,922 | 1,065 |

**Table 6. Correcting for polymerase slippage.** ‘Total sites (with ambiguous sites)’ is how many sites were identified after accounting for the 9 bp duplication associated with Tn10 insertion events. ‘Sites remaining after removing ambiguous sites’ is how many sites remained after removing adjacent sites with the same read count (in most cases less than or equal to 3), for which a local maximum could not be un-ambiguously assigned. ‘Sites remaining after correcting for slippage’ is how many sites remained after collapsing read counts for adjacent sites onto the site with the highest count (local maximum). ‘Total sites removed’ is how many sites were removed when correcting for slippage.

| Condition | Replicate | Max:min |
| --- | --- | --- |
| Input | 1 | 2.30 |
| Anoxic | 1 | 1.58 |
|  | 2 | 1.56 |
| Oxic | 1 | 1.53 |
|  | 2 | 1.49 |
| Mono-infection | 1 | 1.43 |
|  | 2 | 1.38 |
| Co-infection | 1 | 1.44 |
|  | 2 | 1.43 |

**Table 7. Correcting for distance from the origin.** ‘Max:min’ is the fold difference between the maximum and minimum value of the LOESS regression, indicating how much insertions close to the origin were inflated relative to those close to the terminus.

**Figure 1. Removing bias related to gene length.** Left: Read counts per gene are dependent on gene length. Right: This bias was removed after applying normalization factors calculated by EDASeq (6).

***TdT/2-PCR method for preparing Tn-seq Illumina libraries***

***Overview***


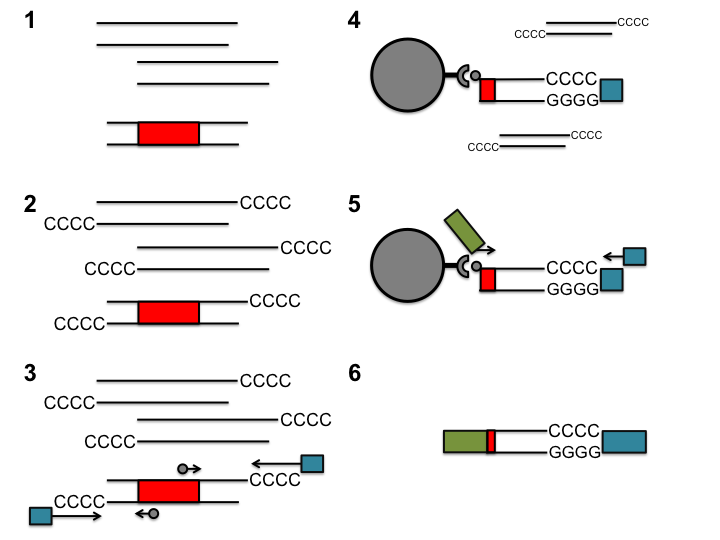
(1) Genomic DNA from a transposon mutant pool is extracted and sheared. Only some of the fragments contain a transposon insert (red). (2) A C-tail is added to the 3’ end of each fragment using the TdT enzyme, providing a universal priming site. (3) PCR-1: A biotinylated primer (grey) targets the transposon (red). A C-tail-specific primer appends the Illumina Read 2 primer site (blue). (4) Biotinylated products from PCR-1 are purified from background genomic DNA using streptavidin-coupled DynaBeads. (5) PCR-2: A second transposon-specific primer builds the entire P5 Illumina adapter/flow cell capture site and the Read 1 primer site (green). The other primer targets the Read 2 primer site and contains the library-specific barcode and the P7 Illumina adapter/flow cell capture site (blue). (6) The final product contains both the Illumina P5 and P7 adapters and Read 1 and Read 2 primer sites for sequencing. See accompanying PowerPoint for complete details.

***References***

-Klein *et al.*, 2012 (*BMC Genomics*) <http://www.biomedcentral.com/1471-2164/13/578>

-Goodman *et al.*, 2011 (*Nature Protocols*) <http://www.nature.com/nprot/journal/v6/n12/abs/nprot.2011.417.html>

-Mintz, 2004 (*Microbiology*) <http://mic.sgmjournals.org/content/150/8/2677.short>

-Jacobs *et al.*, 2003 (*PNAS*) <http://www.pnas.org/content/100/24/14339.short>

-Bae *et al.*, 2004 (*PNAS*) <http://www.pnas.org/content/101/33/12312.short>

***Materials***

-recombinant terminal deoxynucleotidyl transferase (rTdT) (Promega, cat. no. M1875)

-5x rTdT reaction buffer

-dCTP/ddCTP mixture

-dCTP, 9.5 mM

-ddCTP (VWR, cat. no. 101172-042), 0.5 mM

-Expand Long Template DNA polymerase (Roche, cat. no. 11681842001) (see note 1)

-Expand Long Template Buffer 2, 10x concentrated

-dNTPs mixture, 10 mM

-DynaBeads® M-280 Streptavidin (Life Technologies, cat. no. 11205D)

-2x binding & wash (B&W) buffer

-2M NaCl

-10 mM Tris-HCl

-1 mM EDTA, pH 7.5

-low TE (LoTE) buffer

-3 mM Tris-HCl

-0.2 mM EDTA, pH 7.5

-AMPure XP beads (Beckman Coulter, cat. no. A63880)

-magnetic separation rack (NEB, cat. no. S1506S)

-spin columns for DNA purification

-tube rotator

-thermocycler

-molecular biology-grade H_2_O

-DNase-free Eppendorf tubes

-barrier pipette tips

***Primer design***

*Tn-1*

The Tn-1 primer should be 5’-biotinylated. If possible, Tn-1 should be designed to amplify in only one direction off the transposon.

*Tn-2*

The Tn-2 primer should hybridize downstream of Tn-1 but *not* at the very edge of the transposon. Instead, Tn-2 should be nested at least 10-15 bases into the transposon. This is for bioinformatics purposes. Primers can be non-specific, and these 10-15 bases serve as a way to bioinformatically distinguish sequencing reads that are derived from your transposon from those that are present because of offsite priming. The Tn-2 primer is very long and should be PAGE purified (an option that should be available when the primer is ordered). The five random bases (N’s) are meant to help with generating cluster diversity on the Illumina flow cell. Please e-mail us if you have any questions!

*Barcode (BC) primers*

These primers are identical except for a 6 bp ‘barcode’ sequence. These barcodes give each library a unique ID and allow them to be sequenced on the same Illumina flow cell. Labs that plan to do many Tn-seq experiments should have several barcode primers. Barcode sequences can be found here: <https://wikis.utexas.edu/display/GSAF/Illumina+-+all+flavors>. Since these are reverse primers, please note to use the *reverse complement* of what is listed on the website (see table below for examples).

The melting temperatures (T_m_) in the tables listed below were calculated using the default settings in OligoAnalyzer 3.1 (<https://www.idtdna.com/calc/analyzer>) and correspond to the underlined regions.

*Transposons*

| Transposon | Plasmid | Organism | Reference |
| --- | --- | --- | --- |
| Tn10 | pVT1542 | *Aggregatibacter actinomycetemcomitans* | Mintz, 2004 (*Microbiology*) |
| T8 (Tn5 derviative) | pIT2 | *Pseudomonas aeruginosa* | Jacobs *et al.*, 2003 (*PNAS*) |
| mariner | pMR361-K* | *Aggregatibacter actinomycetemcomitans* | Bae *et al.*, 2004 (*PNAS*) |
| *This is a mariner delivery plasmid constructed by Dr. Matthew Ramsey. | | | |

*PCR-1 primers*

| Primer | Description | Sequence (5’->3’) | T_m_ |
| --- | --- | --- | --- |
| *Forward* | | | |
| Tn-1 | biotinylated; targets the Tn | biotin-(insert your sequence) |  |
| Tn10-1 | Tn-1 primer for Tn10 | biotin-TTTACACTGATGAATGTTCCGTTGCGCTGC | 63ºC |
| T8-1 | Tn-1 primer for T8 | biotin-GGGTTTTCCCAGTCACGACGTTG | 61ºC |
| mariner-1 | Tn-1 primer for mariner | biotin-ACTCACTATAGGAGGGCGGGAATCATTTGAAGGTTGGTAC |  |
| *Reverse* | | | |
| olj376 | targets the C-tail; adds the Read 2 primer site | GTGACTGGAGTTCAGACGTGTGCTCTTCCGATCTGGGGGGGGGGGGGGGG | 72ºC |

*PCR-2 primers*

| Primer | Description | Sequence (5’->3’) | T_m_ |
| --- | --- | --- | --- |
| *Forward* | | | |
| Tn-2 | targets the Tn; adds the P5 adapter and Read 1 primer site | AATGATACGGCGACCACCGAGATCTACACTCTTTCCCTACACGACGCTCTTCCGATCTNNNNN-  (insert your sequence) |  |
| Tn10-2 | Tn-2 primer for Tn10 | AATGATACGGCGACCACCGAGATCTACACTCTTTCCCTACACGACGCTCTTCCGATCTNNNNN  AGATGTGTATCCACCTTAACTTAATGATTTTTACC | 57ºC |
| T8-2 | Tn-2 primer for T8 | AATGATACGGCGACCACCGAGATCTACACTCTTTCCCTACACGACGCTCTTCCGATCTNNNNN  CGTCCAGGACGCTACTTGTG | 58ºC |
| mariner-2 | Tn-2 primer for mariner | AATGATACGGCGACCACCGAGATCTACACTCTTTCCCTACACGACGCTCTTCCGATCTNNNNN  GTGTCAGACCGGGGACTTATCAG | 59ºC |
| *Reverse* | | | |
| BC-- | barcode primers; add the library-specific barcode and the P7 adapter | CAAGCAGAAGACGGCATACGAGATxxxxxxGTGACTGGAGTTCAGACGTGTG | 58ºC |
| BC39 | primer with the TruSeq 39 barcode | CAAGCAGAAGACGGCATACGAGATGTATAGGTGACTGGAGTTCAGACGTGTG | 58ºC |
| BC40 | primer with the TruSeq 40 barcode | CAAGCAGAAGACGGCATACGAGATTCTGAGGTGACTGGAGTTCAGACGTGTG | 58ºC |
| BC42 | primer with the TruSeq 42 barcode | CAAGCAGAAGACGGCATACGAGATCGATTAGTGACTGGAGTTCAGACGTGTG | 58ºC |

***Protocol***

1. Shear genomic DNA to a 100-700 bp size range (ideally 200-400 bp). We use a QSonica 800R with the settings given below, but for some *in vivo* samples, we have found that it helps to further size-select the DNA using AMPure beads (see note 2 for AMPure bead size selection protocol). This ensures that all of the DNA that goes through the Tn-seq protocol is exactly within the target size range, especially small fragments which may be preferentially amplified (PCR bias) or large fragments which may inhibit Illumina bridge amplification. Sheared DNA should be run out on a gel to determine its size range (see note 3 for example gels).

| QSonica 800R settings for shearing DNA to 200-400 bp | |
| --- | --- |
| amplitude | 60% |
| pulse rate | 10 seconds on |
|  | 10 seconds off |
| total sonication on time | 10 minutes |
| water bath temperature | 4ºC |
| sample volume | ≥250 µl |

2. Set up the TdT tailing reaction:

| Reagent | Volume (µl) |
| --- | --- |
| sheared DNA (2.5 µg) | - |
| 9.5 mM dCTP / 0.5 mM ddCTP | 2.5 |
| 5x rTdT reaction buffer | 10 |
| rTdT | 1.25 |
| H_2_O | to 50 µl |

3. Incubate the reaction at 37ºC for 1 hour.

4. Purify either with AMPure beads (see step 12 for protocol) or with spin columns (follow the manufacturer’s protocol; if given the option, add isopropanol). Elute in 25 µl H_2_O.

5. Set up PCR-1:

| Reagent | Volume (µl) |
| --- | --- |
| tailed template (250-500ng) | - |
| 10x Buffer 2 | 5 |
| dNTPs (10 mM) | 2.5 |
| Tn-1 primer (30 µM) | 1 |
| olj376 primer (30 µM) | 3 |
| DNA polymerase | 0.75 |
| H_2_O | up to 50 µl |

6. Run the following thermocycler program:

| Temperature | Time |  |
| --- | --- | --- |
| 95ºC | 5 minutes |  |
| 94ºC | 30 seconds | 10x |
| 60ºC* | 30 seconds |  |
| 68ºC | 2 minutes |  |
| 68ºC | 10 minutes |  |
| 16ºC | hold |  |

*The annealing temperature may be different depending on the design of your Tn-1 primer. The temperature shown here has been successful in making libraries with our Tn10-1, T8-1, and mariner-1 primers.

7. Purify (either with AMPure beads or columns). Elute in 50 µl H_2_O.

8. Bind purified product to Dynabeads:

-resuspend beads, add the following volume to a 1.5 ml Eppendorf tube:

32 µl x the total number of reactions

-on magnetic stand, wash beads 3x with 1 ml 1x B&W buffer

-remove final wash, resuspend beads in the following volume of 2x B&W buffer:

52 µl x the total number of reactions

-add 50 µl purified product to 50 µl beads, rotate at room temperature for 30 minutes

-wash 1x with 100 µl 1x B&W buffer, 2x with 100 µl LoTE, remove final wash

9. Set up PCR-2 by resuspending the beads in the following reaction mixture:

| Reagent | Volume (µl) |
| --- | --- |
| PCR-1 product | DynaBeads |
| 10x Buffer 2 | 5 |
| dNTPs (10 mM) | 2.5 |
| Tn-2 primer (30 µM) | 1 |
| BC-- primer (30 µM) | 1 |
| DNA polymerase* | 0.75 |
| H_2_O | 39.75 |

*To avoid shearing the polymerase, add it to the PCR-2 mixture after the DynaBeads are fully resuspended.

10. Run the following themocycler program:

| Temperature | Time |  |
| --- | --- | --- |
| 95ºC | 5 minutes |  |
| 94ºC | 30 second | 15x |
| 58ºC* | 30 seconds |  |
| 68ºC | 2 minutes^#^ |  |
| 68ºC | 10 minutes |  |
| 16ºC | hold |  |

*The annealing temperature may be different depending on the design of your Tn-2 primer. The temperature shown here has been successful in making libraries with our Tn10-2, T8-2, and mariner-2 primers.

^#^We recommend gently flicking the tube at the 1-minute mark of each 68ºC extension segment to keep the DynaBeads in solution.

11. On a magnetic stand, collect the supernatant from the beads and transfer it to a new tube. Purify the supernatant with 40 µl AMPure beads (see protocol below). Elute in a small volume (10-15 µl) H_2_O. Do not use spin columns here since they will not remove adapter contamination.

1. Adjust sample volume to 50 µl with H_2_O
2. Add a 0.8X ratio of AMPure beads (40 µl)
3. Mix well by thoroughly pipetting and/or gently vortexing
4. Incubate at room temperature for 15 minutes
5. Place tube on magnetic stand, let suspension clear
6. Remove supernatant, add 200 µl 80% ethanol, wait 30 seconds
7. Remove ethanol, add 200 µl 80% ethanol, wait 30 seconds
8. Carefully remove all ethanol, let dry on magnetic stand for 10 minutes

*Do not over-dry the sample as this can lead to lower DNA recovery

1. Take tube off magnetic stand, resuspend in elution volume of H_2_O
2. Incubate at room temperature for 5 minutes
3. Place tube back on magnetic stand, let suspension clear
4. Transfer supernatant to new tube

12. At this point, we submit our samples to the core facility at UT-Austin for quality control, including a Bioanalyzer run (see note 4 for example Bioanalyzer results) and qPCR.

*Troubleshooting:*

-If the Bioanalyzer shows that the library has adapter contamination, re-purify it with 0.8X AMPure beads.

-If the library is not detectable on the Bioanalyzer, you should still request for qPCR data because your sample may still contain reads. The qPCR will accurately determine the number of reads your library will yield from Illumina sequencing.

-If your sample does not contain library according to qPCR, we recommend increasing the C-tailed template in PCR-1 to 2 µg (or the remaining DNA from the C-tailing reaction). If that does not work, try scaling up the TdT reaction to ≥10 µg and adding all of it to PCR-1.

-If a library is detectable and lacks adapters, then it is ready to be sequenced!

***Notes***

1. We have had the most success constructing Tn-seq libraries using the Expand Long Template system.

2. AMPure bead size selection protocol

1. Remove large fragments (>700 bp)
   1. Adjust sample volume to 50 µl with H_2_O
   2. Add 0.5X beads (25 µl), mix thoroughly
   3. Incubate at room temperature for 15 minutes
   4. Place sample on magnetic stand, let suspension clear
   5. Remove 72 µl supernatant, place in a new tube

*The supernatant contains your sample, the fragments below 700 bp

1. Remove small fragments (<100 bp)
   1. To the 72 µl supernatant, add 0.7X beads (50.4 µl), mix thoroughly
   2. Incubate at room temperature for 15 minutes
   3. Place sample on magnetic stand, let suspension clear
   4. Remove supernatant and discard (optional: save supernatant at -20^o^C in case of error)

*Your sample is now on the beads; the supernatant contains small fragments <100 bp

1. With sample on magnetic stand, add 200 µl 80% ethanol
2. Incubate sample for 30 seconds, discard ethanol
3. Repeat steps 3 and 4 (for two total 80% ethanol washes)
4. Let sample air dry on magnetic stand at room temperature for 10 minutes
5. Remove sample from stand, resuspend in elution volume of H_2_O
6. Incubate sample at room temperature for 5 minutes
7. Place sample back on magnetic stand, let suspension clear
8. Transfer clear supernatant to new tube (remove 2 µl less than elution volume added to avoid beads)

3a. Gel of a sonicated DNA sample before size selection (1 kb plus ladder):


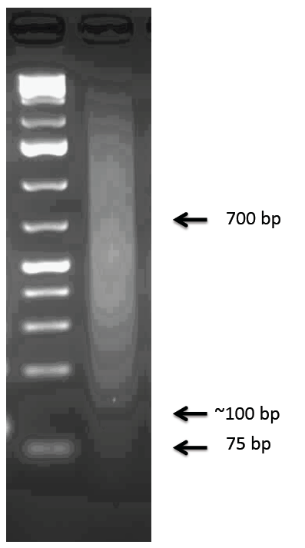


3b. Gel of a size-selected DNA sample that a Tn-seq library was made from (1 kb plus ladder):


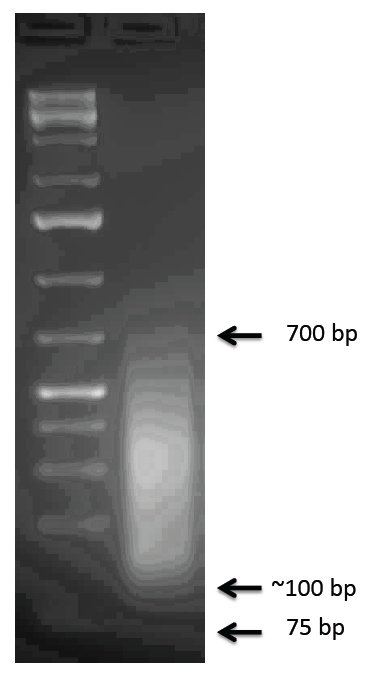


4a. Bioanalyzer result for a library that has adapter contamination:


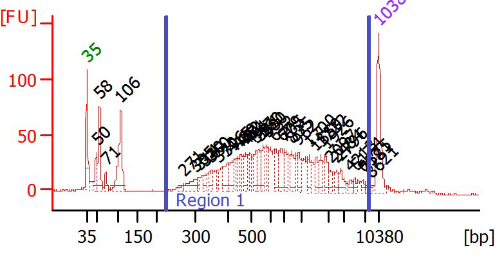


4b. Bioanalyzer result for a library that was sequenced:


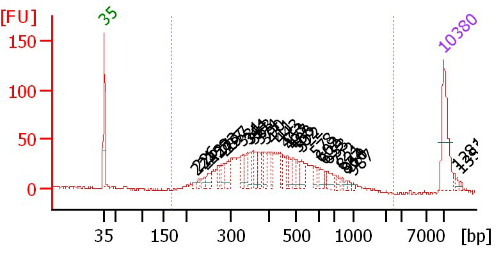


4c. Bioanalyzer result for another library that was sequenced. Libraries are ideally in the 300-500 bp range but can still be sequenced if they have peaks much larger than that. What is key is that they do not have adapter contamination since adapters will consume a large fraction of your sequencing reads.


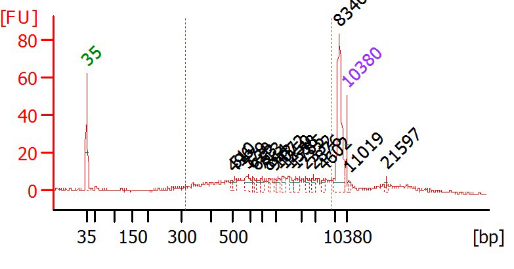


***Modified INSeq method for preparing Tn-seq Illumina libraries***

***Reference***

Goodman *et al.*, 2011 (*Nature Protocols*) <http://www.nature.com/nprot/journal/v6/n12/abs/nprot.2011.417.html>

***Materials***

-Expand Long Template DNA polymerase (Roche, cat. no. 11681842001)

-Expand Long Template Buffer 2, 10x concentrated

-dNTPs mixture, 10 and 25 mM

-DynaBeads® M-280 Streptavidin (Life Technologies, cat. no. 11205D)

-2x binding & wash (B&W) buffer

-2M NaCl

-10 mM Tris-HCl

-1 mM EDTA, pH 7.5

-low TE (LoTE) buffer

-3 mM Tris-HCl

-0.2 mM EDTA, pH 7.5

-AMPure XP beads (Beckman Coulter, cat. no. A63880)

-magnetic separation rack (NEB, cat. no. S1506S)

-spin columns for DNA purification

-tube rotator

-thermal cycler

-molecular biology-grade H_2_O

-DNase-free Eppendorf tubes

-barrier pipette tips

-NEBuffers, BSA

-random primers (Invitrogen, cat. no. 48190-011)

-Klenow fragmet (3’->5’ exo^-^) (NEB, cat. no. M0212S)

-NotI-HF (NEB, cat. no. R3189S)

-NEBNext DNA Library Prep Master Mix Set for Illumina (NEB, cat. no. E6040S)

-T4 DNA ligase (400,000 U/ml) (NEB, cat. no. M0202S)

***Primer design***

*INSeq capture primer*

This primer should have the following design (5’->3’): biotin label, NotI restriction site, transposon-specific sequence. The transposon-specific sequence should be nested several bases (>10) from the end of the transposon. These bases provide a control sequence in downstream analyses to ensure that sequencing reads are derived from the transposon.

| Name | Sequencing (5’->3’) | Underlined region |
| --- | --- | --- |
| INSeq-Tn10 | biotin-GGCCGCGATTTTTACCAAAATCATTAGG | NotI restriction site |
| Illumina primer 1 (P5) | AATGATACGGCGACCACCGAGA |  |
| Illumina primer 2 (P7) | ATCTCGTATGCCGTCTTCTGCTTG |  |

1. Set up 2 100-µl linear PCR reactions:

| Reagent | Volume (µl) |
| --- | --- |
| sheared DNA (0.5-1 µg) | - |
| 10x Buffer 2 | 10 |
| dNTPs (10 mM) | 2 |
| INSeq-Tn10 (1 pmol/µl) | 5 |
| DNA polymerase | 0.75 |
| H_2_O | to 100 µl |

2. Run the following thermal cycler program:

| Temperature | Time | Cycles |
| --- | --- | --- |
| 94ºC | 2 minutes |  |
| 94ºC | 15 seconds | 99x |
| 55ºC | 30 seconds |  |
| 68ºC | 2 minutes |  |
| 68ºC | 7 minutes |  |
| 16ºC | hold |  |

3. Pool the reactions, purify with a spin column, elute in 50 µl H_2_O.

4. Bind purified product to Dynabeads:

-resuspend beads, add the following volume to a 1.5 ml Eppendorf tube:

32 µl x the total number of reactions

-on magnetic stand, wash beads 3x with 1 ml 1x B&W buffer

-remove final wash, resuspend beads in the following volume of 2x B&W buffer:

52 µl x the total number of reactions

-add 50 µl purified product to 50 µl beads, rotate at room temperature for 30 minutes

-wash 1x with 100 µl 1x B&W buffer, 2x with 100 µl LoTE

Pause point: Sample can be stored in 100 µl LoTE at 4ºC overnight.

5. Set up the second strand synthesis reaction:

| Reagent | Volume (µl) |
| --- | --- |
| 10x NEBuffer 2 (or 4) | 2 |
| random primers | 1.375-2 |
| 10 mM dNTPs | 1 |
| H_2_O | to 19 µl |

6. Collect the beads on magnetic stand, discard supernatant, resuspend in second strand synthesis reaction.

7. Denature the DNA by incubating in a thermal cycler for 2 minutes at 95ºC, quickly chill in an ice-water bath.

8. Add 1 µl Klenow fragment (3’->5’ exo^-^), mix gently.

9. Incubate in a thermal cycler for 30 minutes at 37ºC, gently flick every 10-15 minutes.

9. Wash the beads 3x with 100 µl LoTE.

Pause point: Sample can be stored in 100 µl LoTE at 4ºC overnight.

10. Set up the NotI-HF digest reaction:

| Reagent | Volume (µl) |
| --- | --- |
| 10x NEBuffer 4 | 2 |
| 10x BSA (10 µg/ml) | 2 |
| H_2_O | 15 µl |

11. Resuspend beads in the NotI-HF reaction, add 1 µl NotI-HF.

12. Incubate in a thermal cycler for 1 hour at 37ºC, gently flick every 10-15 minutes.

13. Inactivate the NotI-HF by incubating in a thermal cycler for 20 minutes at 75ºC.

14. Collect the supernatant (the beads can be discarded).

15. Set up the end repair reaction:

| Reagent | Volume (µl) |
| --- | --- |
| NotI-digested DNA | 20 |
| NEBNext End Repair Reaction Buffer (10x) | 10 |
| NEBNext End Repair Enzyme Mix | 5 |
| H_2_O | 65 |

16. Incubate in a thermal cycler for 30 minutes at 20ºC.

17. Purify with a spin column, elute in 42 µl H_2_O.

18. Set up the dA-tailing reaction:

| Reagent | Volume (µl) |
| --- | --- |
| End Repaired, Blunt DNA | 42 |
| NEBNext dA-Tailing Reaction Buffer (10x) | 5 |
| Klenow Fragment (3’->5’ exo^-^) | 3 |

19. Incubate in a thermal cycler for 30 minutes at 37ºC.

20. Purify with a spin column, elute in 32.8 µl H_2_O.

21. Set up the adaptor ligation reaction:

| Reagent | Volume (µl) |
| --- | --- |
| dA-tailed DNA | 32.8 |
| Illumina barcode adaptor* (5 µM) | 1.2 |
| 10x T4 DNA ligase reaction buffer | 4 |
| T4 DNA ligase | 2 |
| For details: <https://wikis.utexas.edu/display/GSAF/Illumina+-+all+flavors> | |

22. Incubate in a thermal cycler for 2 hours (or overnight) at 16ºC.

23. Purify 2x with AMPure beads (elute second purification in 42.05 µl H_2_O):

1. Adjust sample volume to 50 µl with H_2_O
2. Add a 0.8X ratio of AMPure beads (40 µl)
3. Mix well by thoroughly pipetting and/or gently vortexing
4. Incubate at room temperature for 15 minutes
5. Place tube on magnetic stand, let suspension clear
6. Remove supernatant, add 200 µl 80% ethanol, wait 30 seconds
7. Remove ethanol, add 200 µl 80% ethanol, wait 30 seconds
8. Carefully remove all ethanol, let dry on magnetic stand for 10 minutes

*Do not over-dry the sample as this can lead to lower DNA recovery

1. Take tube off magnetic stand, resuspend in elution volume of H_2_O
2. Incubate at room temperature for 5 minutes
3. Place tube back on magnetic stand, let suspension clear
4. Transfer supernatant to new tube

24. Set up the PCR enrichment reaction:

| Reagent | Volume (µl) |
| --- | --- |
| Adaptor Ligated DNA | 42.05 |
| 10x Buffer 2 | 5 |
| 25 mM dNTPs | 1 |
| 25 µM Illumina primer 1 (P5) | 0.6 |
| 25 µM Illumina primer 2 (P7) | 0.6 |
| DNA polymerase | 0.75 |

25. Run the following thermal cycler program:

| Temperature | Time | Cycles |
| --- | --- | --- |
| 94ºC | 2 minutes |  |
| 94ºC | 15 seconds | 18x |
| 65ºC | 30 seconds |  |
| 68ºC | 2 minutes |  |
| 68ºC | 7 minutes |  |

26. Purify 1x AMPure beads, elute in 10-15 µl H_2_O.

27. Analyze by Bioanalyzer and qPCR.

***Spectrophotometric assay for measuring TMAO***

***Reference***

Anderson & Allen, 2011 (Journal of Fish Biology)

<http://onlinelibrary.wiley.com/doi/10.1111/j.1095-8649.2010.02875.x/full>

***Materials***

-FeSO_4_-EDTA reagent (0.1 M FeSO_4_, 0.1 M disodium EDTA in 0.8 M acetate buffer)

-0.2 M FeSO_4_

-dissolve 2.78 g FeSO_4_•7H_2_O in a small volume of diH_2_O

-add 0.415 ml concentrated HCl (~0.1 N HCl final solution)

-dilute to 50 ml; discard once no longer color-less

-0.2 M EDTA

-dissolve 14.89 g Na_2_EDTA•2H_2_O in 200 ml diH_2_O

-add 10 M NaOH (5-7 ml, adjusts pH to >8) until completely dissolved

-Fe-EDTA reagent

-calibrate pH meter; add following components to a small beaker with a stir bar:

-(1) 0.5 ml glacial acetic acid, (2) 5 ml FeSO_4_, (3) 5 ml EDTA

-adjust pH to 4.5-4.6 with 10 M NaOH (>1 ml)

-use within 1 hour (discard if turns deep yellow-brownish red)

-0.8 M acetate buffer

-dilute 2 ml glacial acetic acid to 40 ml with diH_2_O

-adjust pH to 4.5-4.6 with 10 M NaOH

-toluene

-solvent-resistant polypropylene 96-well plate

-45% (w/v) KOH solution

-0.2% picric acid in toluene

*2% stock solution (stable for at least 1 year)*

-dissolve 2.9 g picric acid (containing ~30% water) in 100 ml toluene

-store at room temperature in brown bottle away from light

*0.2% working solution (make fresh daily)*

-dilute 1 ml stock solution with 9 ml toluene

-media blanks

-incubate under same conditions as cultures (e.g. 37ºC for 24 hours)

-standards

-0.05, 0.1, 0.5, 1 mM TMA/O diluted in diH_2_O (or media if comparing to cultures)

***Procedure***

1. Pre-heat heat block (with wells filled with H_2_O) to 50ºC; prepare blanks, standards, and Fe-EDTA reagent.

2a. TMA + TMAO analysis:

-per sample, add to a microcentrifuge tube:

-(1) 300 µl FeSO_4_-EDTA, (2) 300 µl sample, (3) 300 µl toluene

2b. TMA analysis:

-per sample, add to a microcentrifuge tube:

-(1) 300 µl acetate buffer, (2) 300 µl sample, (3) 300 µl toluene

3. Incubate samples for 5 min at 50ºC.

4. Cool to room temperature for 1 min, briefly centrifuge.

5. Add 100 µl 45% KOH solution.

6. Vortex at max speed for 30 sec, briefly centrifuge.

7. Per sample, add 200 µl 0.2% picric acid to a well of a solvent-resistant polypropylene 96-well plate.

8. Transfer 100 µl toluene phase from each sample to corresponding well, mix by pipetting.

9. Measure at 410 nm on a spectrophotometric plate reader.

***Script for processing/mapping Aa 2-PCR libraries***

(modified from TnSeq2.sh available at <https://github.com/khturner/Tn-seq>)

#!/usr/bin/env bash

#example code for how to run script from the command line:

#TnSeq2-Aa.sh -p NNNNNAGATGTGTATCCACCTTAACTTAATGATTTTTACC -i AAAATCATTAGGGGATTCATCAG -a VT1169 -m 1 filename

#This requires fqgrep (https://github.com/indraniel/fqgrep)

usage () {

echo "usage: $0 [-p <primer seq>] [-i <IR seq>] [-a <assembly>] <pfx> "

echo "Required parameters:"

echo "-p The sequence of your Tn-seq primer specific to your transposon"

echo "-i The sequence of the transposon end sequence remaining (for junction authentication)"

echo "-a The name of the assembly you're using (PAO1)"

echo "-m The number of mismatches/indels you want to tolerate during search"

echo ""

echo "The required parameters must precede the file prefix for your sequence file:"

echo " (e.g. if your sequence file is named condition1_R1.fastq,"

echo " the prefix is \"condition1\")"

echo ""

echo "Example:"

echo "$0 -p CGTCCAGGACGCTACTTGTG -i TATAAGAGTCAG -a PAO1 -m 1 condition1"

}

# Read in the important options

while getopts ":p:i:a:m:" option; do

case "$option" in

p) PRIMER="$OPTARG" ;;

i) IR="$OPTARG" ;;

a) ASSEMBLY="$OPTARG" ;;

m) MISMATCHES="$OPTARG" ;;

h) # it's always useful to provide some help

usage

exit 0

;;

:) echo "Error: -$option requires an argument"

usage

exit 1

;;

?) echo "Error: unknown option -$option"

usage

exit 1

;;

esac

done

shift $(( OPTIND - 1 ))

# Do some error checking to make sure parameters are defined

if [ -z "$PRIMER" ]; then

echo "Error: you must specify the primer sequence using -p"

usage

exit 1

fi

if [ -z "$IR" ]; then

echo "Error: you must specify the Tn end sequence using -i"

usage

exit 1

fi

if [ -z "$ASSEMBLY" ]; then

echo "Error: you must specify an assembly using -a"

usage

exit 1

fi

if [ -z "$MISMATCHES" ]; then

echo "Error: you must specify a number of mismatches using -m"

usage

exit 1

fi

# Give the usage if there aren't enough parameters

if [ $# -lt 1 ] ; then

echo "you must provide a file prefix for analysis"

usage

exit 1

fi

PREFIX=$1

R1=${PREFIX}_R1

BOWTIEREF=ref_genome/$ASSEMBLY/$ASSEMBLY

echo "Performing TnSeq analysis on $PREFIX..."

echo "TnSeq processing stats for $PREFIX" > $PREFIX-TnSeq.txt

echo "Total sequences: " >> $PREFIX-TnSeq.txt

egrep -c '^@HWI|^@M|^@NS|^@SRR' $R1.fastq >> $PREFIX-TnSeq.txt

# IRs

echo "$PREFIX: Searching for reads with an IR in right location..."

fqgrep -m $MISMATCHES -r -p $IR $R1.fastq | awk -F "\t" '(($8 >= 62 && $8 <= 64) || $1=="read name")' | trimmer --5-prime > $PREFIX-IR-clip.fastq

~/.local/bin/cutadapt -q 20 -a C{16} -m 20 $PREFIX-IR-clip.fastq > $PREFIX-IR-clip.trim.fastq 2> $PREFIX-cutadapt-report.txt

mv $PREFIX-IR-clip.trim.fastq $PREFIX-IR-clip.fastq

IRSFOUND=$(egrep -c '^@HWI|^@M' $PREFIX-IR-clip.fastq)

echo "Processed sequences:" >> $PREFIX-TnSeq.txt

echo $IRSFOUND >> $PREFIX-TnSeq.txt

# Map and convert - feel free to change bowtie2 parameters yourself

echo "$PREFIX: Mapping with Bowtie2..."

echo "Bowtie2 report:" >> $PREFIX-TnSeq.txt

bowtie2 --end-to-end -p 16 -x $BOWTIEREF -U $PREFIX-IR-clip.fastq -S $PREFIX.sam 2>> $PREFIX-TnSeq.txt

grep '^@' $PREFIX.sam > $PREFIX-mapped.sam

cat $PREFIX.sam | grep -v '^@' | awk -F "\t" '((and($2, 0x4) != 0x4) && ($5 > 39))' >> $PREFIX-mapped.sam

echo "Number of reads mapping at high enough MAPQ:" >> $PREFIX-TnSeq.txt

grep -v '^@' $PREFIX-mapped.sam | wc -l >> $PREFIX-TnSeq.txt

echo "$PREFIX: Tallying mapping results..."

grep -v '^@' $PREFIX-mapped.sam | awk -F "\t" '{if (and($2, 0x10) != 0x10) print $4; else print $4+length($10)-9}' | grep '[0-9]' | sort | uniq -c | sort -n -r > $PREFIX-sites.txt

echo "Number of insertion sites identified:" >> $PREFIX-TnSeq.txt

wc -l $PREFIX-sites.txt >> $PREFIX-TnSeq.txt

echo "Most frequent sites:" >> $PREFIX-TnSeq.txt

head $PREFIX-sites.txt >> $PREFIX-TnSeq.txt

#Generate IGV files

echo "$PREFIX: Generating files for IGV..."

samtools view -b -S $PREFIX-mapped.sam > $PREFIX-mapped.bam 2> /dev/null

samtools sort $PREFIX-mapped.bam $PREFIX-mapped.sorted > /dev/null

samtools index $PREFIX-mapped.sorted.bam > /dev/null

# Sort output, cleanup

echo "$PREFIX: Cleaning up..."

mkdir $PREFIX 2> /dev/null

mv $PREFIX-TnSeq.txt $PREFIX/

mv $PREFIX-IR-clip.fastq $PREFIX/

mv $PREFIX.sam $PREFIX/

mv $PREFIX-mapped.sam $PREFIX/

mv $PREFIX-mapped.bam $PREFIX/

mv $PREFIX-sites.txt $PREFIX/

mkdir $PREFIX/IGV 2> /dev/null

mv $PREFIX-mapped.sorted.bam $PREFIX/IGV

mv $PREFIX-mapped.sorted.bam.bai $PREFIX/IGV

mv $PREFIX-cutadapt-report.txt $PREFIX/

***Script for processing/mapping Aa INSeq libraries***

(modified from TnSeq2.sh available at <https://github.com/khturner/Tn-seq>)

#!/usr/bin/env bash

#example code for how to run script from the command line:

#INSeq-Aa.sh -p GGCCGCGATTTTTACCAAAATCATTAGG -i GGATTCATCAG -a VT1169 -m 0 filename

#This requires fqgrep (https://github.com/indraniel/fqgrep)

usage () {

echo "usage: $0 [-p <primer seq>] [-i <IR seq>] [-a <assembly>] <pfx> "

echo "Required parameters:"

echo "-p The sequence of your Tn-seq primer specific to your transposon"

echo "-i The sequence of the transposon end sequence remaining (for junction authentication)"

echo "-a The name of the assembly you're using (PAO1)"

echo "-m The number of mismatches/indels you want to tolerate during search"

echo ""

echo "The required parameters must precede the file prefix for your sequence file:"

echo " (e.g. if your sequence file is named condition1_R1.fastq,"

echo " the prefix is \"condition1\")"

echo ""

echo "Example:"

echo "$0 -p CGTCCAGGACGCTACTTGTG -i TATAAGAGTCAG -a PAO1 -m 1 condition1"

}

# Read in the important options

while getopts ":p:i:a:m:" option; do

case "$option" in

p) PRIMER="$OPTARG" ;;

i) IR="$OPTARG" ;;

a) ASSEMBLY="$OPTARG" ;;

m) MISMATCHES="$OPTARG" ;;

h) # it's always useful to provide some help

usage

exit 0

;;

:) echo "Error: -$option requires an argument"

usage

exit 1

;;

?) echo "Error: unknown option -$option"

usage

exit 1

;;

esac

done

shift $(( OPTIND - 1 ))

# Do some error checking to make sure parameters are defined

if [ -z "$PRIMER" ]; then

echo "Error: you must specify the primer sequence using -p"

usage

exit 1

fi

if [ -z "$IR" ]; then

echo "Error: you must specify the Tn end sequence using -i"

usage

exit 1

fi

if [ -z "$ASSEMBLY" ]; then

echo "Error: you must specify an assembly using -a"

usage

exit 1

fi

if [ -z "$MISMATCHES" ]; then

echo "Error: you must specify a number of mismatches using -m"

usage

exit 1

fi

# Give the usage if there aren't enough parameters

if [ $# -lt 1 ] ; then

echo "you must provide a file prefix for analysis"

usage

exit 1

fi

PREFIX=$1

R1=${PREFIX}_R1

BOWTIEREF=ref_genome/$ASSEMBLY/$ASSEMBLY

echo "Performing TnSeq analysis on $PREFIX..."

echo "TnSeq processing stats for $PREFIX" > $PREFIX-TnSeq.txt

echo "Total sequences: " >> $PREFIX-TnSeq.txt

egrep -c '^@HWI|^@M|^@NS|^@SRR' $R1.fastq >> $PREFIX-TnSeq.txt

# IRs

echo "$PREFIX: Searching for reads with an IR in right location..."

fqgrep -m $MISMATCHES -r -p $IR $R1.fastq | awk -F "\t" '(($8 >= 37 && $8 <= 43) || $1=="read name")' | trimmer --5-prime > $PREFIX-IR-clip.fastq

~/.local/bin/cutadapt -q 20 -a AGATCGGAAGAGC -m 20 $PREFIX-IR-clip.fastq > $PREFIX-IR-clip.trim.fastq 2> $PREFIX-cutadapt-report.txt

mv $PREFIX-IR-clip.trim.fastq $PREFIX-IR-clip.fastq

PROCESSED=$(egrep -c '^@HWI|^@M' $PREFIX-IR-clip.fastq)

echo "Processed sequences:" >> $PREFIX-TnSeq.txt

echo $PROCESSED >> $PREFIX-TnSeq.txt

# Map and convert - feel free to change bowtie2 parameters yourself

echo "$PREFIX: Mapping with Bowtie2..."

echo "Bowtie2 report:" >> $PREFIX-TnSeq.txt

bowtie2 --end-to-end -p 16 -x $BOWTIEREF -U $PREFIX-IR-clip.fastq -S $PREFIX.sam 2>> $PREFIX-TnSeq.txt

grep '^@' $PREFIX.sam > $PREFIX-mapped.sam

cat $PREFIX.sam | grep -v '^@' | awk -F "\t" '((and($2, 0x4) != 0x4) && ($5 > 39))' >> $PREFIX-mapped.sam

echo "Number of reads mapping at high enough MAPQ:" >> $PREFIX-TnSeq.txt

grep -v '^@' $PREFIX-mapped.sam | wc -l >> $PREFIX-TnSeq.txt

echo "$PREFIX: Tallying mapping results..."

grep -v '^@' $PREFIX-mapped.sam | awk -F "\t" '{if (and($2, 0x10) != 0x10) print $4; else print $4+length($10)-9}' | grep '[0-9]' | sort | uniq -c | sort -n -r > $PREFIX-sites.txt

echo "Number of insertion sites identified:" >> $PREFIX-TnSeq.txt

wc -l $PREFIX-sites.txt >> $PREFIX-TnSeq.txt

echo "Most frequent sites:" >> $PREFIX-TnSeq.txt

head $PREFIX-sites.txt >> $PREFIX-TnSeq.txt

#Generate IGV files

echo "$PREFIX: Generating files for IGV..."

samtools view -b -S $PREFIX-mapped.sam > $PREFIX-mapped.bam 2> /dev/null

samtools sort $PREFIX-mapped.bam $PREFIX-mapped.sorted > /dev/null

samtools index $PREFIX-mapped.sorted.bam > /dev/null

# Sort output, cleanup

echo "$PREFIX: Cleaning up..."

mkdir $PREFIX 2> /dev/null

mv $PREFIX-TnSeq.txt $PREFIX/

mv $PREFIX-IR-clip.fastq $PREFIX/

mv $PREFIX.sam $PREFIX/

mv $PREFIX-mapped.sam $PREFIX/

mv $PREFIX-mapped.bam $PREFIX/

mv $PREFIX-sites.txt $PREFIX/

mkdir $PREFIX/IGV 2> /dev/null

mv $PREFIX-mapped.sorted.bam $PREFIX/IGV

mv $PREFIX-mapped.sorted.bam.bai $PREFIX/IGV

mv $PREFIX-cutadapt-report.txt $PREFIX/

***Script for correcting polymerase slippage***

import sys

sites = {}

with open(sys.argv[1], 'U') as f:

for line in f:

l = line.split("\t")

try:

sites[int(l[0])] = int(l[1])

except ValueError:

1+1

id = 0

next_id = 0

results = {}

for s in sorted(sites):

if s <= id:

continue

if sites.get(s, -1) == 0:

continue

local_max_id = s

local_max = sites[s]

sum = sites[s]

id = s + 1

while sites.get(id) > -1:

sum = sum + sites.get(id)

if sites.get(id) > local_max:

local_max = sites.get(id)

local_max_id = id

id = id + 1

results[local_max_id] = sum

for r in sorted(results):

print(str(r) + "\t" + str(results[r]))

**References**

1. Mintz KP & Fives-Taylor PM (2000) impA, a gene coding for an inner membrane protein, influences colonial morphology of *Actinobacillus actinomycetemcomitans*. *Infect Immun* 68(12):6580-6586.

2. Mintz KP (2004) Identification of an extracellular matrix protein adhesin, EmaA, which mediates the adhesion of *Actinobacillus actinomycetemcomitans* to collagen. *Microbiol* 150(8):2677-2688.

3. Brogan JM, Lally ET, & Demuth DR (1996) Construction of pYGK, an *Actinobacillus actinomycetemcomitans-Escherichia coli* shuttle vector. *Gene* 169(1):141-142.

4. Ramsey MM, Rumbaugh KP, & Whiteley M (2011) Metabolite cross-feeding enhances virulence in a model polymicrobial infection. *PLoS Pathog* 7(3):e1002012.

5. Wang Y, Goodman SD, Redfield RJ, & Chen C (2002) Natural transformation and DNA uptake signal sequences in *Actinobacillus actinomycetemcomitans*. *J Bacteriol* 184(13):3442-3449.

6. Risso D, Schwartz K, Sherlock G, & Dudoit S (2011) GC-content normalization for RNA-Seq data. *BMC Bioinformatics* 12:480.
