# Supplementary figures and images for: A Commensal Bacterium Promotes Virulence of an Opportunistic Pathogen via Cross-Respiration
Source: mBio. 2016 Jun 28;7(3):e00782-16. doi: 10.1128/mBio.00782-16 (PMC4916382; doi:10.1128/mBio.00782-16)

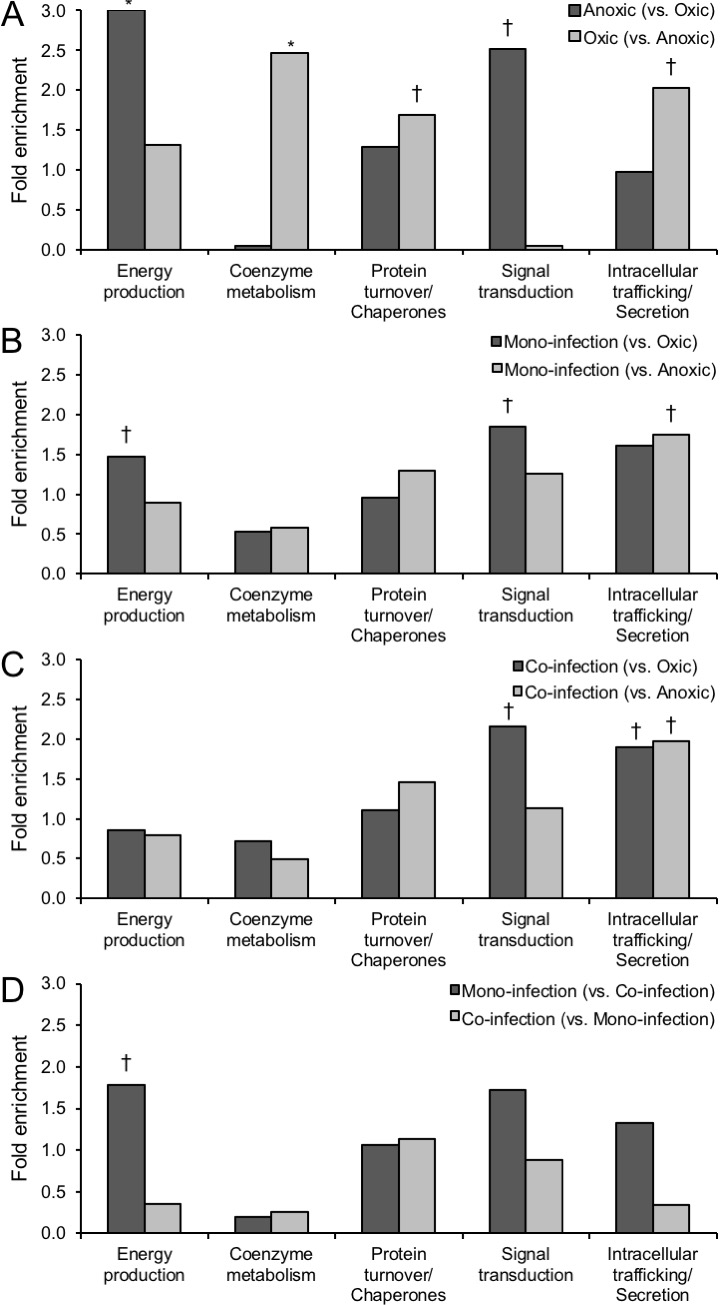

Supplement: Figure S1 — Enrichment of Clusters of Orthologous Groups (COGs) among fitness determinants. Download [file mbo003162854sf1.jpg]

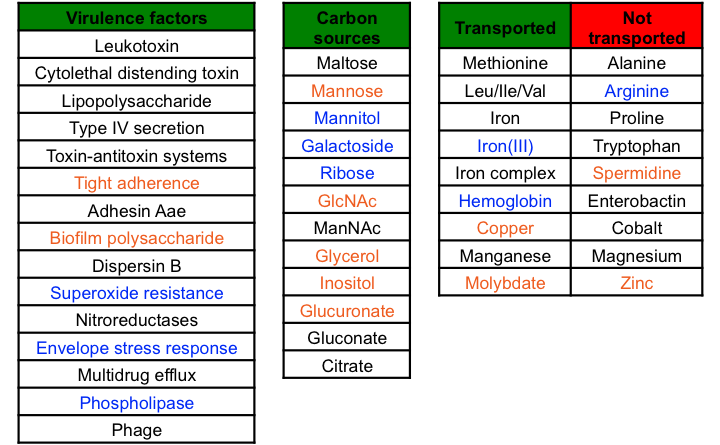

Supplement: Figure S2 — Requirements for monoinfection. Download [file mbo003162854sf2.tif]

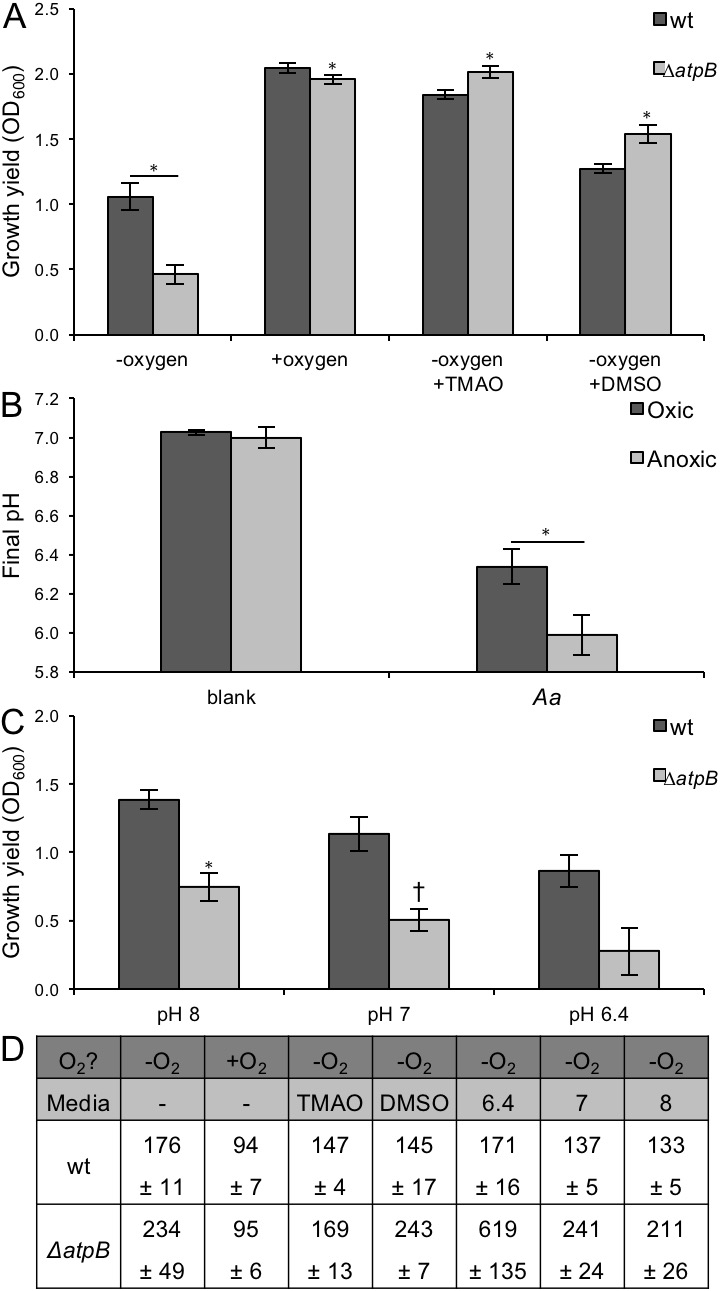

Supplement: Figure S3 — The A. actinomycetemcomitans ATP synthase mutant is rescued by providing electron acceptors or buffering the pH. Download [file mbo003162854sf3.jpg]

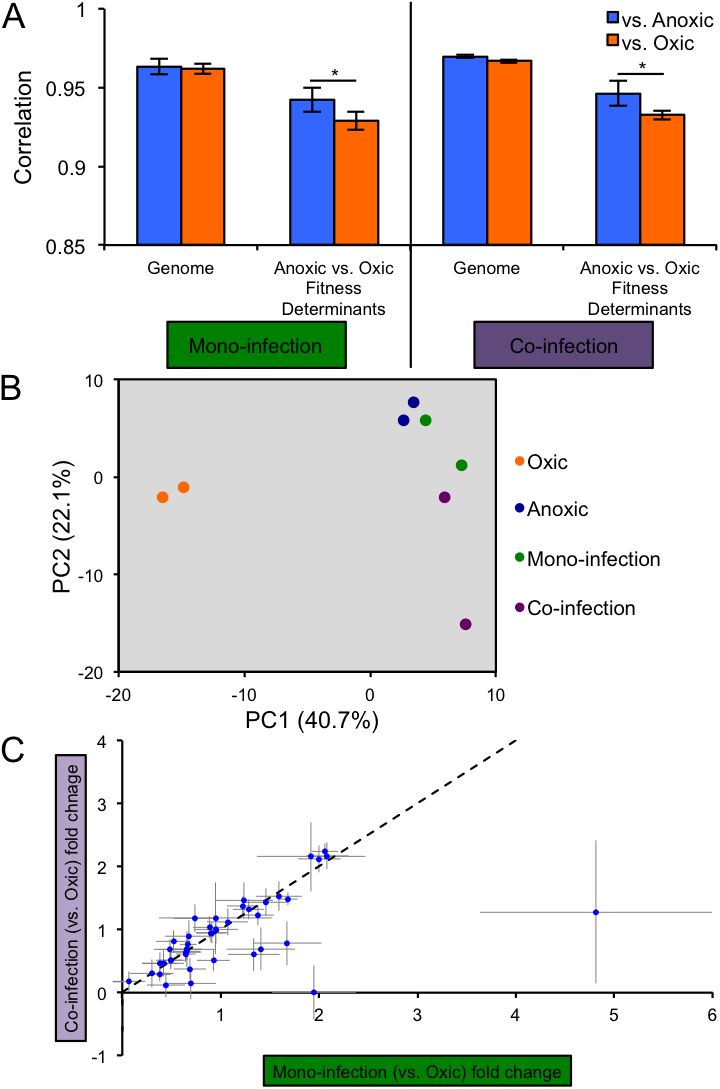

Supplement: Figure S4 — Coinfection shifts A. actinomycetemcomitans away from anoxic growth. Download [file mbo003162854sf4.tif]

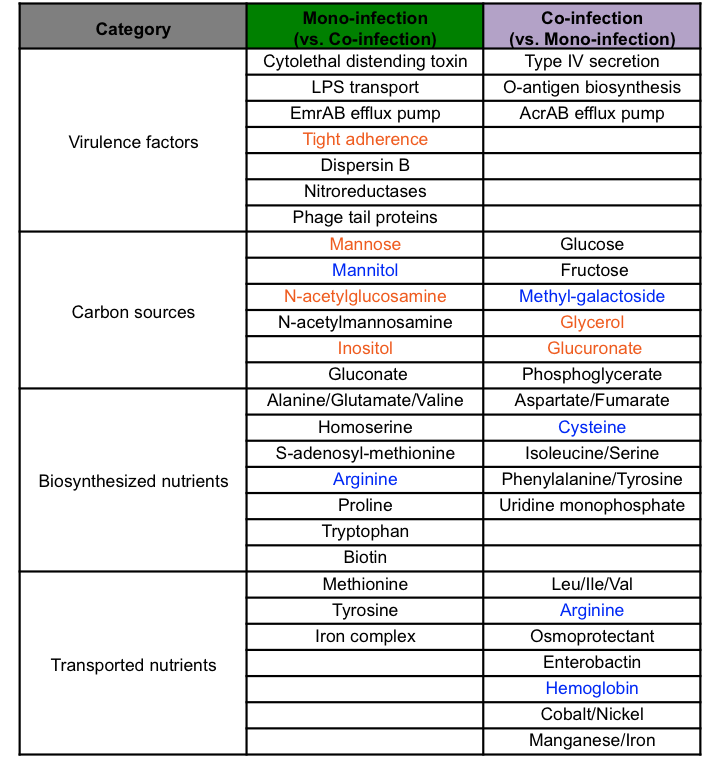

Supplement: Figure S5 — Requirements for mono- and coinfection. Download [file mbo003162854sf5.tif]

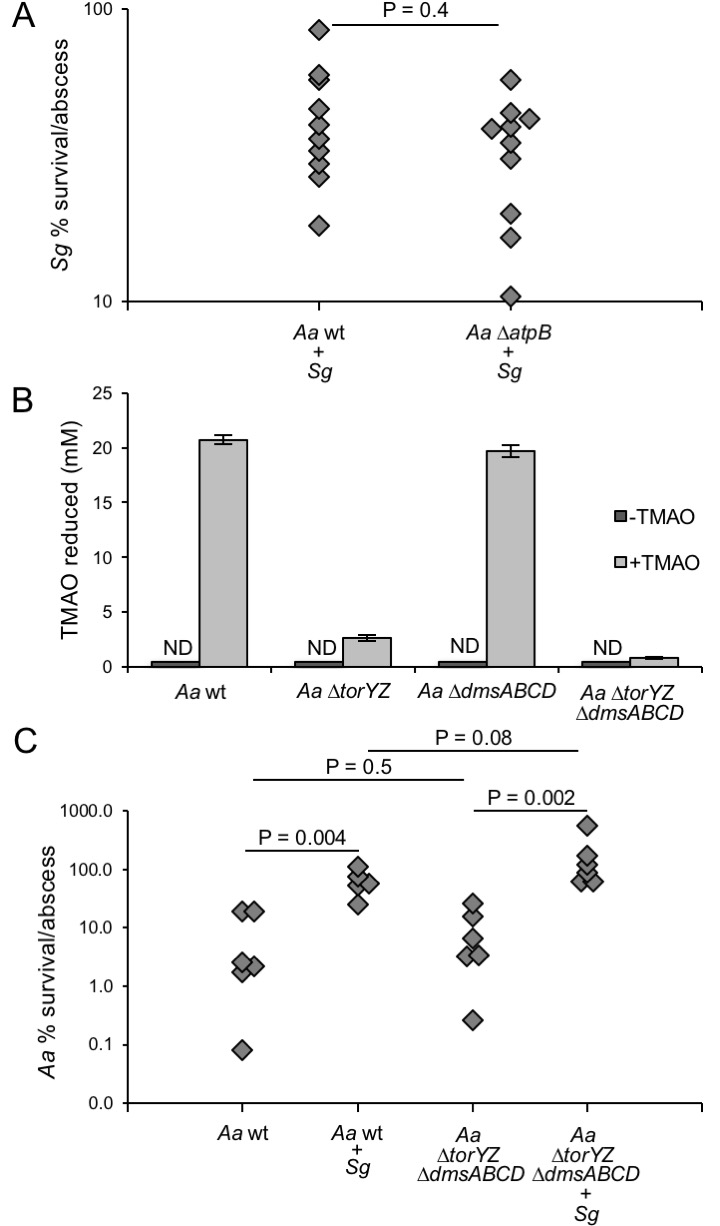

Supplement: Figure S6 — Virulence of S. gordonii and the A. actinomycetemcomitans TMAO/DMSO reductase mutant. Download [file mbo003162854sf6.jpg]
