# Supplementary material for: A Commensal Bacterium Promotes Virulence of an Opportunistic Pathogen via Cross-Respiration
Source: mBio. 2016 Jun 28;7(3):e00782-16. doi: 10.1128/mBio.00782-16 (PMC4916382; doi:10.1128/mBio.00782-16)
Supplement: Dataset S1 — Tables summarizing fitness determinants identified in Tn-seq experiments. Download [file mbo003162854sd1.docx]

| **Category** | **Fitness determinants for Anoxic (vs. Oxic) growth *in vitro*** | **Fitness determinants for Oxic (vs. Anoxic) growth *in vitro*** |
| --- | --- | --- |
| Virulence factors |  | Leukotoxin^T^ (1/1) |
|  |  | Lipopolysaccharide (O-antigen biosynthesis)^B^ (3/3) |
|  |  | Tight adherence^B^ (8/8) |
|  |  | Biofilm polysaccharide (poly-N-acetylglucosamine)^B^ (4/4) |
|  | Superoxide resistance (Ferredoxin-NADP reductase)^E^ (1/1) |  |
|  |  | Peroxide resistance (OxyR)^R^ (1/1) |
|  | Envelope stress response (CpxAR two-component system)^R(M)^ (1/1) |  |
|  | Multidrug efflux^T^ (2/2) |  |
|  | Phospholipase^E^ (1/1) |  |
|  |  | Phage tail protein (1/1) |
| Metabolism |  | Glycolysis^E(M)^ (2/2) |
|  | Pyruvate formate lyase^E^ (2/2) |  |
|  | Ethanol fermentation^E^ (1/1) |  |
|  |  | Acetate fermentation^E^ (1/1) |
|  | Formate dehydrogenase (anaerobic)^ET^ (2/6) | Formate dehydrogenase (aerobic)^E^ (4/6) |
|  |  | Hydrogenase^E^ (1/1) |
|  |  | Reductive TCA cycle^T^ (1/1) |
|  |  | Electron transport chain (Quinone biosynthesis)^B(M)^ (1/1) |
|  | ATP synthase^E(M)^ (5/5) |  |
|  | NAD(P) transhydrogenase^E^ (2/2) |  |
|  |  | Acid stress resistance (Sodium:proton antiporter)^T^ (1/1) |
|  | Nitrate respiration^E(M)^ (1/1) |  |
|  | CRP^R^ (1/1) |  |
|  | FNR^R^ (1/1) |  |
| Carbon sources | Maltose^C^ (1/2) | Maltose^C^ (1/2) |
|  | Starch^C^ (1/1) |  |
|  |  | Mannose^C^ (1/1) |
|  | Mannitol^C^ (1/1) |  |
|  | Methyl-galactoside^T^ (1/1) |  |
|  |  | Glucuronate^R^ (1/1) |
|  |  | N-acetylglucosamine^R^ (1/1) |
|  | Ribose^T^ (1/1) |  |
|  |  | Inositol^C^ (1/1) |
|  | Glycerol^C^ (2/6) | Glycerol^TC^ (4/6) |
| Amino acids | Peptidases^E^ (2/3) | Peptidases^E^ (1/3) |
|  | Cysteine^B(M)^ (1/1) |  |
|  |  | Selenocysteine^BO^ (2/2) |
|  | Arginine^T(M)^ (1/1) |  |
|  | Methyl cycle (S-adenosyl-methionine)^B^ (1/2) | Methyl cycle (S-adenosyl-methionine)^B(M)^ (1/2) |
|  | Autoinducer-2^BT^ (2/4) | Autoinducer-2^BT^ (2/4) |
| Nucleotides | Deoxyribonucleotides^B(M)^ (1/2) | Deoxyribonucleotides (1/2) |
| Cofactors, vitamins, and other compounds |  | Nicotinamide adenine dinucleotide (NAD)^BT^ (3/3) |
|  |  | Heme^T(M)^ (1/1) |
|  |  | Glutathione^B(M)O^ (2/2) |
|  |  | Spermidine/Putrescine^T^ (1/1) |
|  |  | Molybdopterin^B^ (9/9) |
| Metals |  | Copper^T^ (1/1) |
|  |  | Iron storage^O^ (1/1) |
|  | Iron(III)^T^ (1/1) |  |
|  | Hemoglobin/Transferrin/Lactoferrin^T^ (1/1) |  |
|  |  | Molybdate^R^ (1/1) |
|  |  | Zinc^T(M)^ (1/1) |

Table 1. Detailed summary of anoxic and oxic fitness determinants. The left column indicates broad categories of fitness determinants (for example, carbon sources). Entries in the middle and right columns indicate specific categories of fitness determinants (for example, maltose, a specific carbon source). Numbers in parentheses indicate how many genes out of the total genes in the same specific category contribute to fitness under anoxic (middle column) or oxic (right column) growth conditions *in vitro*. Superscript letters indicate the specific functions of these fitness determinants: B = biosynthesis; T = transport; C = catabolism; R = regulation; E = enzymatic activity; O = other activity. An M in parentheses next to a superscript indicates that a KEGG module associated with the same function was also disrupted by anoxic or oxic growth (see Dataset S2 for further details). For example, ‘Envelope stress response^R(M)^ (1/1)’ in the anoxic column indicates (a) that 1 of 1 total genes related to the envelope stress response, which is a virulence factor (indicated by the left category column), contribute to fitness under anoxic growth conditions, (b) that this gene is a regulator of the envelope stress response, and finally (c) that the KEGG module for this regulator was also disrupted by anoxic growth. See Dataset S2 (the ‘ox-v-anox (2)’ tab in the spreadsheet) for further details.

| **Category** | **Fitness determinants for Anoxic (vs. Oxic) growth *in vitro*** | **Fitness determinants for Oxic (vs. Anoxic) growth *in vitro*** |
| --- | --- | --- |
| Virulence factors | Superoxide resistance (E) | Leukotoxin transport (T) |
|  | Envelope stress response (R) | O-antigen biosynthesis (B) |
|  | Multidrug efflux (T) | Tight adherence (B) |
|  | Phospholipase (C) | Biofilm polysaccharide (B) |
|  |  | Peroxide resistance (R) |
|  |  | Phage tail protein |
| Metabolism | Pyruvate formate lyase (E) | Glycolysis (E) |
|  | Ethanol fermentation (E) | Acetate fermentation (E) |
|  | Formate dehydrogenase (anaerobic) (E) | Formate dehydrogenase  (aerobic) (E) |
|  | ATP synthase (E) | Electron transport chain (B) |
|  | NAD(P) transhydrogenase (E) | Acid stress resistance (T) |
|  | Nitrate respiration (E) |  |
|  | CRP (R) |  |
|  | FNR (R) |  |
| Carbon sources | Starch (C) | Mannose (C) |
|  | Mannitol (C) | Glucuronate (R) |
|  | Methyl-galactoside (T) | N-acetylglucosamine (R) |
|  | Ribose (T) | Inositol (C) |
|  |  | Glycerol (TC) |
|  |  |  |
| Amino acids | Cysteine (B) | Selenocysteine (B) |
|  | Arginine (T) |  |
| Cofactors, vitamins, and other compounds | Autoinducer-2 signaling (BT) | Nicotinamide adenine dinucleotide (NAD) (BT) |
|  |  | Heme (T) |
|  |  | Glutathione (BO) |
|  |  | Spermidine/Putrescine (T) |
|  |  | Molybdopterin (B) |
|  |  | Autoinducer-2 signaling (BT) |
| Metals | Iron(III) (T) | Copper (T) |
|  | Hemoglobin/Transferrin/Lactoferrin (T) | Iron storage (O) |
|  |  | Molybdate (R) |
|  |  | Zinc (T) |

Table 2. Summary of anoxic and oxic fitness determinants. Letters in parentheses indicate the specific functions of fitness determinants associated with a virulence factor, metabolic pathway/enzyme, or substrate: B = biosynthesis; T = transport; C = catabolism; R = regulation; E = enzymatic activity; O = other activity. For example, we suggest maltose is an important carbon source anaerobically because a gene involved in maltose catabolism was an anoxic fitness determinant. In contrast, we suggest glucuronate is an important carbon source aerobically because a gene involved in the regulation of glucuronate catabolism was an oxic fitness determinant. This table also provides the color scheme that we used to annotate *in vivo* fitness determinants (in the below tables) as either associated with anoxic or oxic stress.

| **Category** | **Fitness determinant in Mono-infection (vs. *in vitro*)** | **Fitness determinants *in vitro* (vs. Mono-infection)** |
| --- | --- | --- |
| Virulence factors | Leukotoxin^M^ (3/3) (1/3)^M^ |  |
|  | Cytolethal distending toxin (1/1) |  |
|  | Lipopolysaccharide (7/15) (1/7) | Lipopolysaccharide (9/15) (3/9) |
|  | Type IV secretion (4/6) | Type IV secretion (2/6) |
|  | Type IV secretion effectors (4/6) (1/4) | Type IV secretion effectors (2/6) |
|  | Tight adherence^B^ (10/11) (7/11) | Tight adherence^B^ (1/11) |
|  | Adhesin Aae (1/1) |  |
|  | Dispersin B^E^ (1/1) |  |
|  | Biofilm polysaccharide (poly-N-acetylglucosamine)^B^ (3/3) |  |
|  | Superoxide resistance^ER^ (2/2) (1/1)^E^ |  |
|  | Nitrotoreductases^E^ (2/2) |  |
|  | Envelope stress response (CpxAR two-component system)^R(M)^ (2/2) (1/2)^R(M)^ |  |
|  | Multidrug efflux^TR^ (4/12) (1/4)^T^ | Multidrug efflux^TR^ (8/12) (1/8)^T^ |
|  | Toxin-antitoxin systems (3/4) | Toxin-antitoxin systems (1/4) |
|  | Alarmone^B^ (2/3) | Alarmone^B^ (1/3) |
|  | Phospholipase^C^ (1/1) |  |
|  | Phage (3/5) (1/3) | Phage (2/5) |
| Metabolism |  | Glycolysis^E(M)^ (2/2) |
|  | Pentose phosphate pathway^E(M)^ (2/4) (oxidative phase, glucose-6P -> ribulose-5P) (gluconate/ribose) | Pentose phosphate pathway^E(M)^ (2/4) (xylose/ribose) |
|  | Pyruvate dehydrogenase/Succinyl-CoA^E(M)^ (1/1) |  |
|  | Pyruvate formate lyase^E^ (1/1) | Pyruvate formate lyase^E^ (1/1) |
|  |  | D-lactate fermentation^E^ (1/1) |
|  |  | Ethanol fermentation^E^ (3/3) (1/3) |
|  | Formate dehydrogenase^E^ (1/7) | Formate dehydrogenase^E^ (6/7) (3/6) (1/6) |
|  |  | Hydrogenase^E^ (6/6) (1/6) |
|  | Reductive TCA cycle^E(M)T^ (2/12) | Reductive TCA cycle^E(M)T^ (10/12) (1/5)^T^ |
|  | Electron transport chain^EBTO^ (4/12) (1/1)^B(M)^ | Electron transport chain^EBT^ (8/12) (1/2)^T^ |
|  | ATP synthase^E(M)^ (3/5) | ATP synthase^E(M)^ (4/5) |
|  | NAD(P) transhydrogenase^E^ (2/2) | NAD(P) transhydrogenase^E^ (2/2) |
|  |  | Acid stress (Sodium:proton antiporter)^T^ (4/4) (1/4) |
|  | Nitrate respiration^R(M)^ (1/3) | Nitrate respiration^E^ (2/3) |
|  |  | DMSO respiration^E^ (3/3) |
|  | CRP^RE^ (1/1)^R^ |  |
|  | FNR^R^ (1/1) |  |
|  |  | CsrA^R^ (1/1) |
|  |  | ArcA^R(M)^ (1/1) |
| Carbon sources |  | Glucose^T^ (1/1) |
|  | Maltose^CR^ (3/6) | Maltose^T(M)C^ (3/6) (1/2)^C^ (1/2)^C^ |
|  | Starch^B(M)^ (1/4) | Starch^B(M)C^ (3/4) (1/2)^C^ |
|  |  | Fructose^T(M)^ (3/3) |
|  | Mannose^C^ (2/6) (1/2) | Mannose^TC^ (5/6) (1/1)^C^ |
|  | Mannitol^T(M)C^ (2/2) (1/1)^C^ |  |
|  | Galactose/Galactoside^T(M)R^ (3/5) (1/2)^T^ | Galactose/Galactoside^TC(M)^ (3/5) (1/1)^T^ |
|  |  | Galactitol^T(M)CR^ (4/4) |
|  | Glucuronate^C(M)R^ (3/3) (1/1)^R^ |  |
|  | Gluconate^T^ (1/2) | Gluconate^R^ (1/2) |
|  | N-acetylglucosamine^CT(M)R^ (3/4) (1/1)^R^ | N-acetylglucosamine^C^ (1/4) |
|  | Other amino sugars^C^ (1/6) | Other amino sugars^C^ (5/6) |
|  | Ribose^TC^ (4/8) | Ribose^TC^ (4/8) (1/3)^T^ |
|  | Xylose^T^ (3/9) | Xylose^T(M)C^ (6/9) (1/3)^C^ |
|  | Glycerol^C^ (7/17) (2/7) (1/7) | Glycerol (12/17)^TC^ (1/9)^C^ (1/9)^C^ (2/3)^T^ |
|  | Citrate^CT^ (2/6) | Citrate^C(M)T^ (5/6) |
|  | Inositol^TC^ (3/8) (1/2)^C^ | Inositol^T(M)C^ (5/8) |
| Amino acids | Peptidases^E^ (1/14) | Peptidases^E^ (13/14) (2/13) (1/13) |
|  |  | Peptide transport^T(M)^ (7/7) |
|  | Alanine^B^ (1/2) | Alanine^T^ (1/2) |
|  |  | Aspartate^B^ (3/3) |
|  |  | Asparagine^B^ (1/1) |
|  | Glutamate^B^ (1/3) | Glutamate^B^ (2/3) |
|  | Glutamine^R^ (1/3) | Glutamine^R^ (2/3) |
|  |  | Serine^B(M)^ (2/2) |
|  | Homoserine^B^ (3/3) |  |
|  | Threonine (from homoserine)^B(M)^ (1/2) | Threonine (from homoserine)^B(M)^ (1/2) |
|  | Cysteine^R^ (1/6) | Cysteine^B(M)O^ (5/6) (1/3)^B^ |
|  | Methionine (from homoserine)^T(M)R^ (2/6) | Methionine (from homoserine)^B(M)^ (4/6) |
|  |  | Selenocysteine^BO^ (6/6) (1/5)^B^ (1/1)^O^ |
|  |  | Methyl cycle (S-adenosyl-methionine)^B^ (2/2) (1/2) |
|  | Autoinducer-2^T^ (3/9) | Autoinducer-2^BTOR^ (6/9) (1/2)^B^ |
|  | Isoleucine^T^ (1/6) | Isoleucine^B(M)T^ (5/6) |
|  | Valine^BT^ (2/6) | Valine^B(M)T^ (4/6) |
|  | Leucine^T^ (1/3) | Leucine^T^ (2/3) |
|  | Arginine^B(M)^ (1/6) | Arginine^B(M)T(M)^ (5/6) (1/4)^T^ |
|  | Proline^B^ (1/3) | Proline^BT^ (2/3) |
|  |  | Phenylalanine^B(M)R^ (3/3) |
|  | Tyrosine^T^ (1/4) | Tyrosine^BR^ (3/4) |
|  |  | Tryptophan^BTR^ (5/5) |
| Nucleotides | Nucleotide salvage^B^ (5/7) | Nucleotide salvage^B^ (2/7) |
|  | Deoxyribonucleotides^B^ (3/13) | Deoxyribonucleotides^M^ (6/13) (1/13) (1/13) |
|  |  | Guanine ribonucleotide^M^ (2/2) |
| Cofactors, vitamins, and other compounds |  | Riboflavin^B(M)R^ (2/2) |
|  |  | Pyridoxal phosphate^B^ (1/1) |
|  | NAD^B^ (1/4) | NAD^BT^ (3/4) (1/3)^T^ (2/3)^B^ |
|  | Biotin^B(M)^ (4/6) | Biotin^B^ (2/6) |
|  | *p*-aminobenzoate^B^ (1/1) |  |
|  | Heme^T(M)^ (1/5) | Heme^B(M)T(M)^ (4/5) (1/2)^T^ |
|  |  | Glutathione^B(M)O^ (3/3) (2/3)^BO^ |
|  |  | Spermidine/Putrescine^T(M)O^ (4/4) (1/2)^T^ |
|  |  | Molybdopterin^B^ (10/10) (9/10)^B^ |
|  | Fumarate^B^ (1/6) | Fumarate^B^ (5/6) |
| Metals |  | Cobalt^T^ (2/2) |
|  | Copper^T^ (1/1) | Copper^T^ (1/1) |
|  | Iron storage^O^ (2/2) (1/2) |  |
|  | Iron^T^ (1/5) | Iron^T^ (5/5) |
|  | Iron(III)^T(M)^ (5/5) (1/5) |  |
|  | Iron complex^T^ (2/7) | Iron complex^T^ (5/7) |
|  |  | Enterobactin^T^ (1/1) |
|  | Hemoglobin/Transferrin/Lactoferrin^T^ (2/3) (1/2) | Hemoglobin/Transferrin/Lactoferrin^T^ (1/3) |
|  |  | Magnesium^T^ (1/1) |
|  | Manganese^T(M)^ (1/3) | Manganese^T(M)^ (3/3) |
|  | Molybdate^TR^ (4/5) (1/1)^R^ | Molybdate^T^ (1/5) |
|  |  | Zinc^T(M)^ (2/2) (1/2) |

Table 3. Detailed summary of mono-infection fitness determinants. The left column indicates broad categories of fitness determinants (for example, carbon sources). Entries in the middle and right columns indicate specific categories of fitness determinants (for example, maltose, a specific carbon source). Numbers in parentheses indicate how many genes out of the total genes in the same specific category contribute to fitness in mono-infection (middle column) or *in vitro* (anoxic and/or oxic growth, right column). Superscript letters indicate the specific functions of these fitness determinants: B = biosynthesis; T = transport; C = catabolism; R = regulation; E = enzymatic activity; O = other activity. An M in parentheses next to a superscript indicates that a KEGG module associated with the same function was also disrupted in mono-infection or *in vitro* (see Dataset S2 for further details). Highlighted entries indicate that all of the associated genes were also fitness determinants for anoxic (highlighted blue) or oxic (highlighted yellow) growth *in vitro*. A highlighted, second set of numbers in parentheses indicates how many genes with a specific function (indicated by the superscript) were also fitness determinants for anoxic (highlighted blue) or oxic (highlighted yellow) growth *in vitro*. For example, ‘Envelope stress response^R(M)^ (2/2) (1/2)^R(M)^’ in the mono-infection column indicates (a) that 2 of 2 total genes related to the envelope stress response, which is a virulence factor (indicated by the left category column), contribute to fitness in mono-infection, (b) that these genes are associated with regulation of the envelop stress response, (c) that the KEGG module for the response was also disrupted in mono-infection, and finally (d) that 1 of the 2 regulators was also a fitness determinant for anoxic growth *in vitro*. See Dataset S2 (the ‘mono (2)’ tab in the spreadsheet) for further details.

| Metabolism  (potentially essential) | Menaquinone/2-Demethyl-Menaquinone |
| --- | --- |
|  | Cytochrome bd complex |
| Amino acid  (potentially essential) | Glycine (from serine) |
|  | Lysine (from aspartate) |
|  | S-adenosyl-methionine |
|  | Chorismate (Shikimate pathway) |
| Amino acids  (potentially auxotrophic) | Histidine (from PRPP) |
|  | Leucine |
| Nucleotides  (potentially auxotrophic) | Inosine monophosphate (from glutamine + PRPP) |
|  | Uridine monophosphate (from glutamine + PRPP) |
| Cofactors, vitamins, and other compounds (potentially essential) | FMN |
|  | FAD |
|  | Coenzyme A (from pantothenate) |
|  | Lipoic acid |
|  | Tetrahydrofolate |
| Cofactors, vitamins, and other compounds (potentially auxotrophic) | Thiamine |
|  | Pantothenate |
|  | Spermidine/Putrescine |

Table 4. Summary of potential *Aa* auxotrophies and essential biosynthetic pathways. Auxotrophies and essential pathways were determined by examining the completeness of pathways in *Aa* VT1169 via the Reconstruct Pathway feature on the KEGG website (<http://www.genome.jp/kegg/tool/map_pathway.html>). Auxotrophies are those where several steps in a pathway appeared to be missing, and essential pathways are those where several steps in a pathway appeared to be essential (that is, 0 mutants detected in any of the tested conditions). PRPP, phosphoribosyl pyrophosphate.

| **Metabolites biosynthesized in**  **Mono-infection (vs. *in vitro*)** | **Metabolites not biosynthesized in**  **Mono-infection (vs. *in vitro*)** |
| --- | --- |
| Alanine (1/1) | Aspartate (3/3) |
| Glutamate (1/3) | Asparagine (1/1) |
| Glutamine (1/3)^R^ | Serine (2/2) |
| Homoserine (3/3) | Cysteine (3/3) |
| Threonine (1/2) | Methionine (4/4) |
| Valine (1/3) | Selenocysteine (5/5) |
| Arginine (1/2) | S-adenosyl-methionine (SAM, methyl cycle) (2/2) |
| Proline (1/2) | Isoleucine (3/3) |
| Nucleotides (salvage pathway) (5/7) | Phenylalanine (2/2) |
| Deoxyribonucleotides (6/9) | Tyrosine (2/2) |
| Nicotinamide adenine dinucleotide (NAD) (1/3) | Tryptophan (3/3) |
| Biotin (4/6) | Guanine ribonucleotide (2/2) |
| *p*-aminobenzoate (PABA) (1/1) | Riboflavin (1/1) |
| Fumarate (1/6) | Pyridoxal phosphate (1/1) |
|  | Heme (3/3) |
|  | Glutathione (1/1) |
|  | Molybdopterin (10/10) |

Table 5. Summary of biosynthetic requirements for mono-infection. Numbers in parentheses indicate how many genes out of the total, differentially fit (in the mono-infection vs. *in vitro* comparison) biosynthetic genes for a metabolite contributed to fitness in mono-infection or *in vitro* (anoxic and/or oxic growth). Metabolites highlighted blue or orange also contributed to fitness under anoxic or oxic growth conditions *in vitro*, respectively (see Table 2). If at least 1 biosynthetic gene for a metabolite was a fitness determinant in mono-infection, that metabolite was considered to be biosynthesized in mono-infection. If all of the differentially fit biosynthetic genes for a metabolite were only fitness determinants *in vitro*, that metabolite was considered to be not biosynthesized in mono-infection. ^R^The biosynthetic genes for glutamine biosynthesis were regulators of the biosynthetic pathway for glutamine rather than enzymes in the pathway. In all other cases, the only genes examined for this analysis were biosynthetic.

| **Nutrients transported in**  **Mono-infection (vs. *in vitro*)** | **Nutrients not transported in**  **Mono-infection (vs. *in vitro*)** |
| --- | --- |
| Methionine (1/1) | Alanine (1/1) |
| Leucine/Isoleucine/Valine (1/3) | Arginine (4/4) |
| Tyrosine (1/1) | Proline (1/1) |
| Iron (1/5) | Tryptophan (1/1) |
| Iron(III) (5/5) | Spermidine/Putrescine (2/2) |
| Iron complex (2/7) | Enterobactin (1/1) |
| Hemoglobin/Transferrin/Lactoferrin (2/3) | Cobalt (2/2) |
| Copper (1/1) | Magnesium (1/1) |
| Manganese (1/3) | Zinc (2/2) |
| Molybdate (3/4) |  |

Table 6. Summary of transport requirements for mono-infection. Numbers in parentheses indicate how many genes out of the total, differentially fit (in the mono-infection vs. *in vitro* comparison) transport genes for a nutrient contributed to fitness in mono-infection or *in vitro* (anoxic and/or oxic growth). Nutrients highlighted blue or orange also contributed to fitness under anoxic or oxic growth conditions *in vitro*, respectively (see Table 2). If at least 1 transport gene for a nutrient was a fitness determinant in mono-infection, that nutrient was considered to be transported in mono-infection. If all of the differentially fit transport genes for a nutrient were only fitness determinants *in vitro*, that nutrient was considered to be not transported in mono-infection.

| **Category** | **Fitness determinants specific to Mono-infection (vs. *in vitro*)** | **Fitness determinants specific to Co-infection (vs. *in vitro*)** |
| --- | --- | --- |
| Virulence factors | Leukotoxin (activation) (1/1) |  |
|  | Cytolethal distending toxin (1/1) |  |
|  | Lipopolysaccharide (transport) (4/5) | Lipopolysaccharide (1/5) |
|  | Tight adherence^B^ (3/3) (1/3) |  |
|  | Dispersin B^E^ (1/1) |  |
|  | Nitroreductases^E^ (2/2) |  |
|  | Multidrug efflux^T^ (2/2) (1/2) |  |
|  | Toxin-antitoxin system (1/2) | Toxin-antitoxin system (1/2) |
|  | Phage tail proteins (2/2) (1/2) |  |
| Metabolism | Pentose phosphate pathway^E^ (1/1) |  |
|  | Pyruvate dehydrogenase^E^/ Succinyl-CoA^B^ (1/1) |  |
|  | Pyruvate formate lyase^E^ (1/1) |  |
|  | Reductive TCA cycle^T^ (1/1) |  |
|  | Electron transport chain^TB^ (cytochrome c biogenesis) (2/3) | Electron transport chain^B^ (cytochrome c biogenesis) (1/3) |
|  | ATP synthase^E^ (2/2) |  |
|  | NAD(P) transhydrogenase^E^ (1/1) |  |
|  | CRP (adenylate cyclase)^E^ (1/1) | CRP^E^ (cAMP phosphodiesterase) (1/1) |
|  | FNR^R^ (1/1) |  |
| Carbon sources |  | Maltose^C^ (2/2) (1/2) |
|  | Starch^B^ (1/1) |  |
|  | Mannose^C^ (1/1) |  |
|  | Mannitol^TC^ (2/2) (1/1)^C^ |  |
|  |  | Glucuronate^C^ (1/1) |
|  | Gluconate^T^ (1/1) |  |
|  | N-acylmannosamine^C^ (1/1) |  |
|  | Ribose^C^ (1/1) |  |
|  | Inositol^TC^ (2/2) (1/1)^C^ |  |
|  |  | Phosphoglycerate^T^ (1/1) |
| Amino acids |  | Lrp |
|  | Alanine/Glutamate/Valine^B^ (1/1) |  |
|  |  | Aspartate/Fumarate^B^ (1/1) |
|  | Glutamine^R^ (1/1) |  |
|  | Homoserine^B^ (1/1) |  |
|  | Cysteine^R^ (1/1) |  |
|  | Methionine (from homoserine)^TR^ (2/2) |  |
|  | Arginine^B^ (1/1) |  |
|  | Proline^B^ (1/1) |  |
| Nucleotides | Nucleotide salvage^B^ (1/1) |  |
|  |  | Uridine monophosphate (UMP)^B^ (1/1) |
| Cofactors, vitamins, and other compounds | Nicotinamide adenine dinucleotide (NAD)^B^ (1/2) | Nicotinamide adenine dinucleotide (NAD)^B^ (1/2) |
|  | Biotin^B^ (1/1) |  |
|  | Heme^T^ (1/1) |  |
|  |  | Osmoprotectant^T^ (2/2) |
| Metals |  | Cobalt/Nickel^T^ (1/1) |
|  | Iron storage^O^ (1/2) | Iron storage^O^ (1/2) |
|  | Iron(III)^T^ (1/2) | Iron(III)^T^ (1/2) |
|  | Iron complex^T^ (1/1) |  |
|  |  | Manganese/Iron^T^ (1/1) |
|  |  | Hemoglobin/Transferrin/Lactoferrin^T^ (1/1) |

Table 7. Detailed summary of fitness determinants that are specific only to mono-infection or only to co-infection, as compared to growth *in vitro*. The left column indicates broad categories of fitness determinants (for example, carbon sources). Entries in the middle and right columns indicate specific categories of fitness determinants (for example, maltose, a specific carbon source). Numbers in parentheses indicate how many genes out of the total genes in the same specific category contribute to fitness specifically in mono-infection (middle column) or co-infection (right column). Superscript letters indicate the specific functions of these fitness determinants: B = biosynthesis; T = transport; C = catabolism; R = regulation; E = enzymatic activity; O = other activity. Highlighted entries indicate that all of the associated genes were also fitness determinants for anoxic (highlighted blue) or oxic (highlighted yellow) growth *in vitro*. A highlighted, second set of numbers in parentheses indicates how many genes with a specific function (indicated by the superscript) were also fitness determinants for anoxic (highlighted blue) or oxic (highlighted yellow) growth *in vitro*. For example, ‘Tight adherence^B^ (3/3) (1/3)’ in the mono-infection column indicates (a) that 3 of 3 total genes related to tight adherence, which is a virulence factor (indicated by the left category column), contribute to fitness specifically in mono-infection, (b) that these genes are associated with the biosynthesis of the tight adherence apparatus, and finally (c) that 1 of the 3 biosynthetic genes was also a fitness determinant for oxic growth *in vitro*. See Dataset S2 (the ‘mono-v-co (specific)’ tab in the spreadsheet) for further details.

| **Category** | **Fitness determinants in Mono-infection (vs. Co-infection)** | **Fitness determinants in Co-infection (vs. Mono-infection)** |
| --- | --- | --- |
| Virulence factors | Leukotoxin activation^M^ (1/2) | Leukotoxin (1/2) |
|  | Cytolethal distending toxin (1/1) |  |
|  | Lipopolysaccharide (transport) (4/6) | Lipopolysaccharide (O-antigen biosynthesis) (2/6) (1/2) |
|  |  | Type IV secretion (1/1) |
|  | Tight adherence^B^ (4/4) (1/4) |  |
|  | Dispersin B^E^ (1/1) |  |
|  | Nitrotoreductases^E^ (2/2) |  |
|  | Multidrug efflux (EmrAB pump)^T(M)^ (2/3) (1/2) | Multidrug efflux (AcrAB pump)^R^ (1/3) |
|  | Toxin-antitoxin systems (3/4) | Toxin-antitoxin systems (1/4) |
|  | Phage tail proteins (2/2) (1/1) |  |
| Metabolism | Pentose phosphate pathway^E^ (1/3) | Pentose phosphate pathway^E^ (2/3) |
|  | Pyruvate dehydrogenase^E(M)^/Succinyl-CoA^B^ (1/1) |  |
|  | Pyruvate formate lyase^E^ (1/1) |  |
|  |  | Formate dehydrogenase (aerobic)^E^ (1/1) |
|  | Reductive TCA cycle^T^ (1/3) | Reductive TCA cycle^E^ (2/3) |
|  | Electron transport chain^T^ (1/3) | Electron transport chain^TB^ (2/3) (1/1)^T^ |
|  | ATP synthase^E(M)^ (4/4) |  |
|  | NAD(P) transhydrogenase^E^ (1/1) |  |
|  |  | DMSO respiration^E^ (1/1) |
|  | TMAO respiration^E^ (1/1) |  |
|  |  | CsrA |
|  | CRP^RE^ (2/3) (1/1)^R^ | CRP^E^ (1/3) |
|  | FNR^R^ (1/1) |  |
| Carbon sources |  | Glucose^T^ (1/1) |
|  | Maltose^T(M)C^ (2/3) (1/1)^C^ | Maltose^C^ (1/3) |
|  | Starch^B(M)^ (1/1) |  |
|  |  | Fructose^T^ (1/1) |
|  | Mannose^C^ (1/1) |  |
|  | Mannitol^T(M)C^ (3/3) (1/1)^C^ |  |
|  |  | Galactose/Galactoside^T(M)C^ (2/2) |
|  | Galactitol^T(M)^ (1/3) | Galactitol^C^ (2/3) |
|  |  | Glucuronate^C(M)^ (1/1) |
|  | Gluconate^T^ (1/1) |  |
|  | N-acetylglucosamine^R^ (1/1) |  |
|  | N-acylmannosamine^C^ (1/1) |  |
|  | Ribose^C^ (1/4) | Ribose^TC^ (3/4) (1/1)^T^ |
|  | Inositol^TC^ (2/2) (1/1)^C^ |  |
|  |  | Glycerol^C^ (3/3) (1/3) |
|  |  | Phosphoglycerate^T(M)^ (2/2) |
| Amino acids |  | Lrp |
|  | Peptidases^E^ (1/2) | Peptidases^E^ (1/2) |
|  | Alanine^B^ (1/1) |  |
|  |  | Aspartate^B^ (1/1) |
|  | Glutamate^B^ (1/1) |  |
|  | Glutamine^R^ (1/2) | Glutamine^R^ (1/2) |
|  |  | Serine^B^ (1/1) |
|  | Homoserine^B^ (1/1) |  |
|  | Cysteine^R^ (1/2) | Cysteine^B(M)^ (1/2) |
|  | Methionine (from homoserine)^T(M)R^ (2/2) |  |
|  | Methyl cycle (S-adenosyl-methionine)^B(M)O^ (2/2) (1/2)^B^ |  |
|  |  | Isoleucine^TB^ (2/2) |
|  | Valine^B^ (1/2) | Valine^T^ (1/2) |
|  | Arginine^B(M)^ (1/2) | Arginine^T(M)^ (1/2) |
|  | Proline^B^ (1/1) |  |
|  |  | Phenylalanine^B^ (1/1) |
|  | Tyrosine^T^ (1/2) | Tyrosine^B^ (1/2) |
|  | Tryptophan^B(M)^ (1/1) |  |
| Nucleotides | Nucleotide salvage^B^ (1/1) |  |
|  |  | Uridine monophosphate^B^ (2/2) |
| Cofactors, vitamins, and other compounds | Nicotinamide adenine dinucleotide (NAD)^B^ (1/2) | Nicotinamide adenine dinucleotide (NAD)^B^ (1/2) |
|  | Biotin^B^ (1/1) |  |
|  | Heme^T(M)^ (1/2) | Heme^T(M)^ (1/2) |
|  |  | Osmoprotectant^T(M)^ (2/2) |
|  |  | Fumarate^B^ (1/1) |
| Metals |  | Cobalt/Nickel^T(M)^ (1/1) |
|  | Iron storage^O^ (1/2) | Iron storage^O^ (1/2) |
|  |  | Iron^T^ (2/2) |
|  | Iron(III)^T^ (2/3) (1/2) | Iron(III)^T^ (1/3) |
|  | Iron complex^T^ (1/1) |  |
|  |  | Enterobactin^T^ (1/1) |
|  |  | Hemoglobin/Transferrin/Lactoferrin^T^ (2/2) |
|  |  | Manganese^T^ (1/1) |

Table 8. Detailed summary of fitness determinants in mono-infection (vs. co-infection) and co-infection (vs. mono-infection). The left column indicates broad categories of fitness determinants (for example, carbon sources). Entries in the middle and right columns indicate specific categories of fitness determinants (for example, maltose, a specific carbon source). Numbers in parentheses indicate how many genes out of the total genes in the same specific category contribute to fitness in mono-infection (middle column) or co-infection (right column). Superscript letters indicate the specific functions of these fitness determinants: B = biosynthesis; T = transport; C = catabolism; R = regulation; E = enzymatic activity; O = other activity. An M in parentheses next to a superscript indicates that a KEGG module associated with the same function was also disrupted in mono-infection or co-infection (see Dataset S2 for further details). Highlighted entries indicate that all of the associated genes were also fitness determinants for anoxic (highlighted blue) or oxic (highlighted yellow) growth *in vitro*. A highlighted, second set of numbers in parentheses indicates how many genes with a specific function (indicated by the superscript) were also fitness determinants for anoxic (highlighted blue) or oxic (highlighted yellow) growth *in vitro*. For example, ‘Multidrug efflux (EmrAB pump)^T(M)^ (2/3) (1/2)’ in the mono-infection column indicates (a) that 2 of 3 total genes related to the EmrAB pump, which is a virulence factor (indicated by the left category column), contribute to fitness in mono-infection, (b) that these genes are associated with transport (the pump itself rather than a regulator), (c) that the KEGG module for the efflux pump was also disrupted in mono-infection, and finally (d) that 1 of the 2 pump components was also a fitness determinant for anoxic growth *in vitro*. See Dataset S2 (the ‘mono-v-co (2)’ tab in the spreadsheet) for further details.

| **Metabolites biosynthesized in**  **Mono-infection (vs. Co-infection)** | **Metabolites biosynthesized in**  **Co-infection (vs. Mono-infection)** |
| --- | --- |
| Ala/Glu/Val (1/1) | Aspartate/Fumarate (1/1) |
| Homoserine (1/1) | Cysteine (1/1) |
| S-adenosyl-methionine (SAM, methyl cycle) (1/1) | Isoleucine/Serine (1/1) |
| Arginine (1/1) | Phenylalanine/Tyrosine (1/1) |
| Proline (1/1) | Uridine monophosphate (UMP, salvage pathway) (2/2) |
| Tryptophan (1/1) |  |
| Biotin (1/1) |  |

Table 9. Summary of biosynthetic requirements in mono- and co-infection. Numbers in parentheses indicate how many genes out of the total, differentially fit (in the mono-infection vs. co-infection comparison) biosynthetic genes for a metabolite contributed to fitness in mono- or co-infection. Metabolites highlighted blue or orange also contributed to fitness under anoxic or oxic growth conditions *in vitro*, respectively (see Table 2). Metabolites with biosynthetic requirements in both mono- and co-infection were not considered.

| **Nutrients transported in**  **Mono-infection (vs. Co-infection)** | **Nutrients transported in**  **Co-infection (vs. Mono-infection)** |
| --- | --- |
| Methionine (1/1) | Leucine/Isoleucine/Valine (1/1) |
| Tyrosine (1/1) | Arginine (1/1) |
| Iron complex (1/1) | Osmoprotectant (2/2) |
|  | Enterobactin (1/1) |
|  | Hemoglobin/Transferrin/Lactoferrin (2/2) |
|  | Cobalt/Nickel (1/1) |
|  | Manganese/Iron (1/1) |

Table 10. Summary of transport requirements in mono- and co-infection. Numbers in parentheses indicate how many genes out of the total, differentially fit (in the mono-infection vs. co-infection comparison) transport genes for a nutrient contributed to fitness in mono- or co-infection. Nutrients highlighted blue or orange also contributed to fitness under anoxic or oxic growth conditions *in vitro*, respectively (see Table 2). Nutrients with transport requirements in both mono- and co-infection were not considered.
